# Effects of an Internet-Based Cognitive Behavioral Therapy (iCBT) Program in Manga Format on Improving Subthreshold Depressive Symptoms among Healthy Workers: A Randomized Controlled Trial

**DOI:** 10.1371/journal.pone.0097167

**Published:** 2014-05-20

**Authors:** Kotaro Imamura, Norito Kawakami, Toshi A. Furukawa, Yutaka Matsuyama, Akihito Shimazu, Rino Umanodan, Sonoko Kawakami, Kiyoto Kasai

**Affiliations:** 1 Department of Mental Health, Graduate School of Medicine, The University of Tokyo, Tokyo, Japan; 2 Departments of Health Promotion and Human Behavior and of Clinical Epidemiology, Graduate School of Medicine/School of Public Health, Kyoto University, Kyoto, Japan; 3 Department of Biostatistics, Graduate School of Medicine, The University of Tokyo, Tokyo, Japan; 4 Kyoto Office, Health Wave Co., Ltd., Kyoto, Japan; 5 Nippon University College of the Arts, Tokyo, Japan; 6 Department of Neuropsychiatry, Graduate School of Medicine, The University of Tokyo, Tokyo, Japan; University Hospital of Bellvitge-IDIBELL; CIBER Fisiopatología Obesidad y Nutrición (CIBERObn), Instituto Salud Carlos III; Department of Clinical Sciences, School of Medicine, University of Barcelona, Spain, Spain

## Abstract

**Objective:**

The purpose of this study was to develop a new Internet-based computerized cognitive behavior therapy (iCBT) program in Manga format, the Japanese cartoon, for workers and to examine the effects of the iCBT program on improving subthreshold depression using a randomized controlled trial (RCT) design among workers employed in private companies in Japan.

**Method:**

All workers in a company (n = 290) and all workers in three departments (n = 1,500) at the headquarters of another large company were recruited by an invitation e-mail. Participants who fulfilled the inclusion criteria were randomly allocated to intervention or control groups (N = 381 for each group). A six-week, six-lesson iCBT program using Manga (Japanese comic) story was developed. The program included several CBT skills: self-monitoring, cognitive restructuring, assertiveness, problem solving, and relaxation. The intervention group studied the iCBT program at a frequency of one lesson per week. Depression (Beck Depression Inventory II; BDI-II) was assessed as a primary outcome at baseline, and three- and six-month follow-ups for both intervention and control groups were performed.

**Results:**

The iCBT program showed a significant intervention effect on BDI-II (t = −1.99, p<0.05) with small effect sizes (Cohen's d: −0.16, 95% Confidence Interval: −0.32 to 0.00, at six-month follow-up).

**Conclusions:**

The present study first demonstrated that a computerized cognitive behavior therapy delivered via the Internet was effective in improving depression in the general working population. It seems critical to improve program involvement of participants in order to enhance the effect size of an iCBT program.

**Trial Registration:**

UMIN Clinical Trials Registry UMIN000006210

## Introduction

Occupational stress has been considered a major risk factor for a wide range of health outcomes [1], [2]. To reduce work-related stress and its negative effect on health among workers, more attention has been paid to the primary prevention strategy. Among other measures, stress management interventions (SMI) have recently become frequently applied in the workplace [3]. A growing body of literature has shown that SMIs based on cognitive behavioral therapy (CBT) [4], [5] and relaxation therapy [6] are effective in reducing work-related stress and improving depression and anxiety among workers. A meta-analysis reported that the estimated effects of CBT and relaxation therapy were Cohen's d = 0.68 and 0.35, respectively [7]. Another meta-analysis reported that the estimated effects of CBT and relaxation therapy were Cohen's d = 1.16 and 0.50, respectively [6]. However, the implementation of these interventions was still limited, because such a program requires professionals well trained in CBT [8]–[10]. In addition, even if a service is provided, the time, cost, and stigma related to mental health treatment could serve as barriers to access to the effective treatment [11].

An innovative way to deliver CBT-based treatment widely is with a computerized CBT (CCBT). CCBT programs can teach information and techniques based on the same CBT principles as face-to-face CBT programs do, with a highly structured format comprising educational lessons, homework assignments, and supplementary resources [12]. Spurgeon and Wright (2010) claimed that there were three major platforms on which to deliver the CCBT programs: 1) multimedia computer programs, 2) virtual reality programs, and 3) Smartphone or handheld device applications. Some programs are delivered in a clinic, and others are accessed at home or through other venues, such as the Internet [13]. The mode of application is an Internet-based cognitive behavioral therapy (iCBT): i.e., a CCBT delivered via the Internet. In addition to the features of CCBT, iCBT has a special feature of high anonymity and high accessibility. In terms of anonymity, iCBT is suitable for addressing psychological problems because it can enable users to avoid the stigma incurred by seeing a therapist [14]. In terms of accessibility, iCBT provides users with the opportunity to obtain treatment at any time and at any place, such as in the workplace or at home, and study the content as much as they want [15]. The level of therapist involvement in iCBT can vary among programs; they could provide no assistance, minimal therapist contact by email or telephone, or a contact similar to face-to-face therapy [15]. However, a complete online iCBT may have merit since it can provide a viable alternate mental health resource for people who have geographical, physical, psychological, and/or financial barriers to seeking traditional, face-to-face care [11].

Previous iCBT programs have been shown to have a significant positive treatment effect on depression and anxiety mostly in the clinical setting. A meta-analysis of RCT reported that iCBT can improve depressive symptoms of patients who have major depressive disorder (Hedges' g = 0.56–0.99) and anxiety disorders (Hedges' g values were 0.92 for social phobia, 0.83 for panic disorder, and 1.12 for generalized anxiety disorder) more effectively compared with TAU or waiting list [16]. The treatment effects of iCBT varied by the extent of therapist involvement. Another meta-analysis reported that the effect size (about 0.6 in Cohen's d on average) of iCBT with therapist support was greater than the effect size (about 0.25) of self-guided program without therapist support [15], [17]. However, these self-guided-only programs showed small to medium effects, and thus a self-guided iCBT program is still considered to be helpful for people who have mild to moderate symptoms but cannot (or do not wish to) see a clinician [12]. Most critical problem within self-guided programs that might have resulted in a smaller effect was high dropout rates [12].

Despite a number of benefits of using CCBT and iCBT, the application of these programs in the workplace setting has been limited. Grime (2004) conducted a randomized controlled trial (RCT) to evaluate the effect of an eight-week CCBT program to improve emotional distress among employees who had recently experienced stress-related absenteeism. A month before the end of the intervention, the depression and anxiety among the participants in the intervention group improved significantly compared to that of the TAU group [18]. Grime (2004) also reported reasons for non-participation [18]. Non-participation in this study was common and related to a number of barriers, such as access problems, preference for other treatments (especially face-to-face counseling), time commitment, skepticism about the intervention, and employer connection. An online or Internet-based CCBT program might be effective in reducing concerns about travel and time off work, confidentiality, and connection with the employer; thus it appears to improve workers' participation more than a CCBT program. However, no iCBT program is available that would target specifically the general working population, the effectiveness of which would be tested with an RCT.

The purpose of this study was to develop a new six-week iCBT program for workers in Japan and to examine the effects of the iCBT program on improving the symptoms of depression at three- and six-month follow-ups using a randomized controlled trial design among workers employed in private companies in Japan. In the iCBT program, we applied a “Manga” (the Japanese cartoon or comic story) format, that is now frequently used to communicate medical information in Japan[19], expecting participants' greater commitment to and better understanding of the contents of the program with using this communicative medium[20], [21].

## Methods

### Trial design

The study was a randomized controlled trial. The allocation ratio of the intervention group to the control group was 1 to 1. The study protocol was registered at the UMIN Clinical Trials Registry (UMIN-CTR) (ID = UMIN000006210). The protocol for this trial and supporting CONSORT checklist are available as supporting information; see Checklist S1, Protocol S1 and Protocol S2.

### Participants

All workers in company A (N = 290) and all workers who belonged to three selected departments (N = about 1,500) at the headquarter of company B (the total employee size, c.a. 11,000) were recruited by an invitation e-mail. Both companies developed information technology systems and related services as their products. Those who were interested in participating in the study were asked to go to a research website to obtain full explanation of the study's aim and procedure and to input their baseline information if they agreed to participate. The inclusion criteria stated that participants could 1) not be diagnosed with a major depressive disorder in the past month (using the web-version of the WHO-Composite International Diagnostic Interview version 3.0 (WHO-CIDI 3.0) [22], 2) not be diagnosed with lifetime bipolar disorder (WHO-CIDI 3.0), 3) not have taken 15 sick leave days or more in total due to their own health problems during the past three months, and 4) not have received medical treatment for mental health problems during the past month.

### Intervention

Participants who were allocated to the intervention group studied the new iCBT program called the *Internet CBT program; Useful mental health solutions series for business*. The program was a six-week web-based training course to provide stress management skills. This program was structured into six lessons, with one lesson given per week. Learning one lesson required about 30 minutes, including homework. This program can be used anywhere the Internet is available.

**Figure 1:**
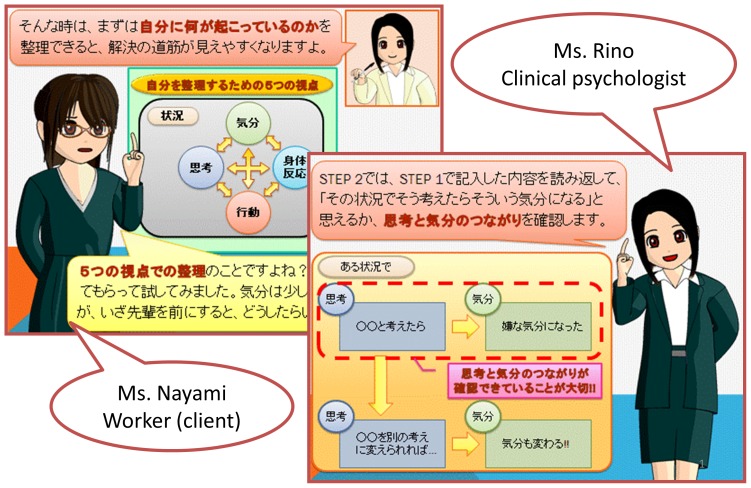
A screen shot of an Internet-based cognitive behavioral therapy (iCBT) program developed in “Manga” (the Japanese cartoon or comic story) format in this study.

One of the unique features of the program was that the training was provided along with a Manga (Japanese comic) story of a psychologist and a client worker to facilitate the understanding of the participants (Figure 1). Several merits of using a comic story and comic characters in education have been acknowledged through research. First, these materials help motivate individuals, and high motivation is useful to keep participants in the program [23]. Second, they facilitate easy learning. A program with text combined with comic stories would be easier for a learner to understand compared to a text-only program [23], [24]. Third, comic stories foster learners' interest in the program [24]. These merits might be applicable to education in the workplace, because most Japanese people of working age are familiar with comics.

In terms of the CBT components of the program, included were self-monitoring skills, cognitive restructuring skills, assertiveness, problem-solving skills, and relaxation skills. At the end of each lesson, participants were asked to submit homework to facilitate their understanding, although it was voluntary. Participants who submitted their homework received feedback from trained staff (clinical psychologists).

The iCBT program included self-monitoring skills (in lesson 2), cognitive restructuring skills (in lessons 3 and 4), assertiveness (in lesson 5), problem-solving skills (in lesson 6), and relaxation skills (in lesson 4). In this study, a cognitive restructuring method was adopted as the primary outcome and main cognitive approach, and this has been shown to be effective in reducing depression [25]. Assertiveness and problem-solving training as well as relaxation skills were chosen as supplementary behavioral approaches to enhance the effect of the program.

### Intervention group

Participants completed six weekly lessons and homework within the iCBT program. They were allowed to complete the six lessons within 10 weeks after the baseline survey. Participants were reminded by e-mail to complete each lesson and/or to submit homework if they had not already done so. Reminders were sent from the research office to the participants every Monday.

### Control group

Participants in the control group received an e-mail message once a month titled “Useful information for stress management.” Each e-mail message consisted of about 500 words in Japanese and included stress management tips. The contents were as follows: 1) How to have a good sleep, 2) The effects of your diet and ingesting alcohol for stress, 3) How to have a good holiday, 4) Relaxing by listening to music and taking a bath, and 5) The effects of exercise on stress. Participants were also able to use an internal employee assistance program service. In addition, an e-learning program on stress and stress management was provided to all employees at company B. This e-learning program contained only a few descriptions of CB-based approaches.

### Outcomes measures

#### Beck Depression Inventory II (BDI-II)

The BDI-II is a 21-item self-report inventory that measures depressive symptoms such as sadness, pessimism, suicidal thoughts or wishes, tiredness or fatigue, loss of energy, and loss of pleasure, among others [26], [27]. Each item is scored on a scale ranging from 0 to 3, with a higher score indicating more serious depressive symptoms.

#### Kessler's psychological distress scale (K6)

Psychological distress was measured with the Japanese version of the K6 scale [28], [29]. K6 consists of six items assessing the frequency with which respondents have experienced symptoms of psychological distress during the past 30 days. The response options range from 0 (none of the time) to 4 (all of the time). The internal reliability and validity found in previous studies are acceptable [28].

#### Japanese version of Dysfunctional Attitude Scale 24 (DAS24-J)

The 24-item Dysfunctional Attitude Scale (DAS-24) is a short version of the Dysfunctional Attitude Scale, which is a self-report inventory measuring depressogenic schemata. Each item is scored on a scale ranging from one (totally disagree) to seven (totally agree), with a higher score indicating a more dysfunctional attitude [30]. The Japanese version has been developed and tested, and its reliability and validity have been established [31].

#### Improvement of knowledge and self-efficacy

Respondents were asked to rate their improvement of knowledge and self-efficacy regarding five components of the iCBT program (Stress management, Cognitive restructuring, Assertive communication, Problem-solving, Relaxation training). Knowledge improvement was assessed by asking participants, “How much knowledge do you have about…” and self-efficacy improvement was assessed by asking respondents “How confident are you that you can do….” Both items were scored on 5-point scale ranging from 0 (none) to 4 (enough).

#### Demographic characteristics

Demographic data, such as age, gender, marital status, occupation, education, chronic disease, and overtime hours during the past month were also collected.

### Sample size calculation

A systematic review of psychological treatments, mostly CBT for subthreshold depression, yielded a Cohen's d of 0.42 (95%CI: 0.23 to 0.60) at post-test [32]. On the other hand, it was reported that the effect of preventive interventions on the incidence of major depressive disorders was smaller in the universal setting than that in the selective or indicated setting [33]. In order to detect an effect size of 0.30 or greater at an alpha error rate of 0.05 and a beta error rate of 0.10, the estimated sample size was 235 participants per arm. With the anticipated dropout rate of 30%, the necessary sample size was 336 participants per arm. The statistical power analysis was conducted using the G*Power 3 program [34], [35].

### Randomization

Participants who fulfilled the inclusion criteria were randomly allocated to intervention or control groups (N = 381 for each group). Stratified permuted-block randomization was conducted. Participants were stratified into four strata according to two factors: K6 score (5 or greater or less than 5) at baseline survey and company (A or B) to which they belonged. A stratified permuted-block random table was generated by an independent biostatistician. Enrollment was conducted by a clinical research coordinator, and assignment was conducted by an independent research assistant. The stratified permuted-block random table was password-protected and blinded to the researcher. Only the research assistant had access to it during the work of random allocation.

### Statistical analyses

A mixed-model for repeated measures conditional growth model analyses were conducted using a group (intervention and control) * time (baseline, three-month, and six-month follow-up) interaction as an indicator of intervention effect. Intention-to-treat analysis (ITT) was conducted. The Linear Mixed Model in the PASW Statistics 18.0 was used. The Number Needed to Treat (NNT) to reduce depressive symptoms or psychological distress to achieve one improvement from subthreshold depression was calculated. Referencing the cutoff scores of BDI-II and K6 of previous studies [26], [36], the improvement was defined to change the scores from 14 or higher to 13 or less on the BDI-II or to change the scores from 5 or higher to 4 or less on the K6. Effect sizes and 95% Confidence Interval (95% CI) were calculated using Cohen's d among those who completed the questionnaire at baseline and at a follow-up. The values of 0.2, 0.5, and 0.8 are generally interpreted as being suggestive of small, medium, and large effects, respectively [37]. Subgroup analyses were conducted separately among respondents who had high (5 or higher score of K6) and low (4 or less score) scores at baseline, with the cutoff score for K6 selected based on a previous report, which suggested that 4/5 is the optimal cutoff point for K6 in terms of discriminating community residents and patients with mood and anxiety disorders [36].

### Ethics

The Research Ethics Review Board of Graduate School of Medicine, the University of Tokyo (No. 3083) approved the study procedures. We prepared a website which contained full explanation of the study. Before the baseline survey, participants were invited to read the explanation on the website, and asked to click an “agree” button to show their consent to participate in the study; then they proceeded to the baseline questionnaire page. Written consent was not required by the National Ethical Guidelines for Epidemiologic Research, Japan; the Research Ethics Review Board of Graduate School of Medicine, the University of Tokyo approved this procedure to obtain participant's consent.

### Changes to the protocol

One major change made to the protocol is the inclusion criteria. Originally it was planned that the participants who scored 5 or more for the K6 on the baseline survey would be allocated. Before the commencement of this study, this criterion was canceled due to the anticipation of a low participation rate. With this, sample size was recalculated. Another change made to the protocol is the method of statistical analysis. Originally the per-protocol analysis was planned, but the ITT analysis was conducted in the present study due to the expectation to be conservative in superiority trials. In addition, NNT was calculated due to the description of the difference between a treatment and a control in achieving a particular outcome.

## Results

### Participant flowchart

**Figure 2:**
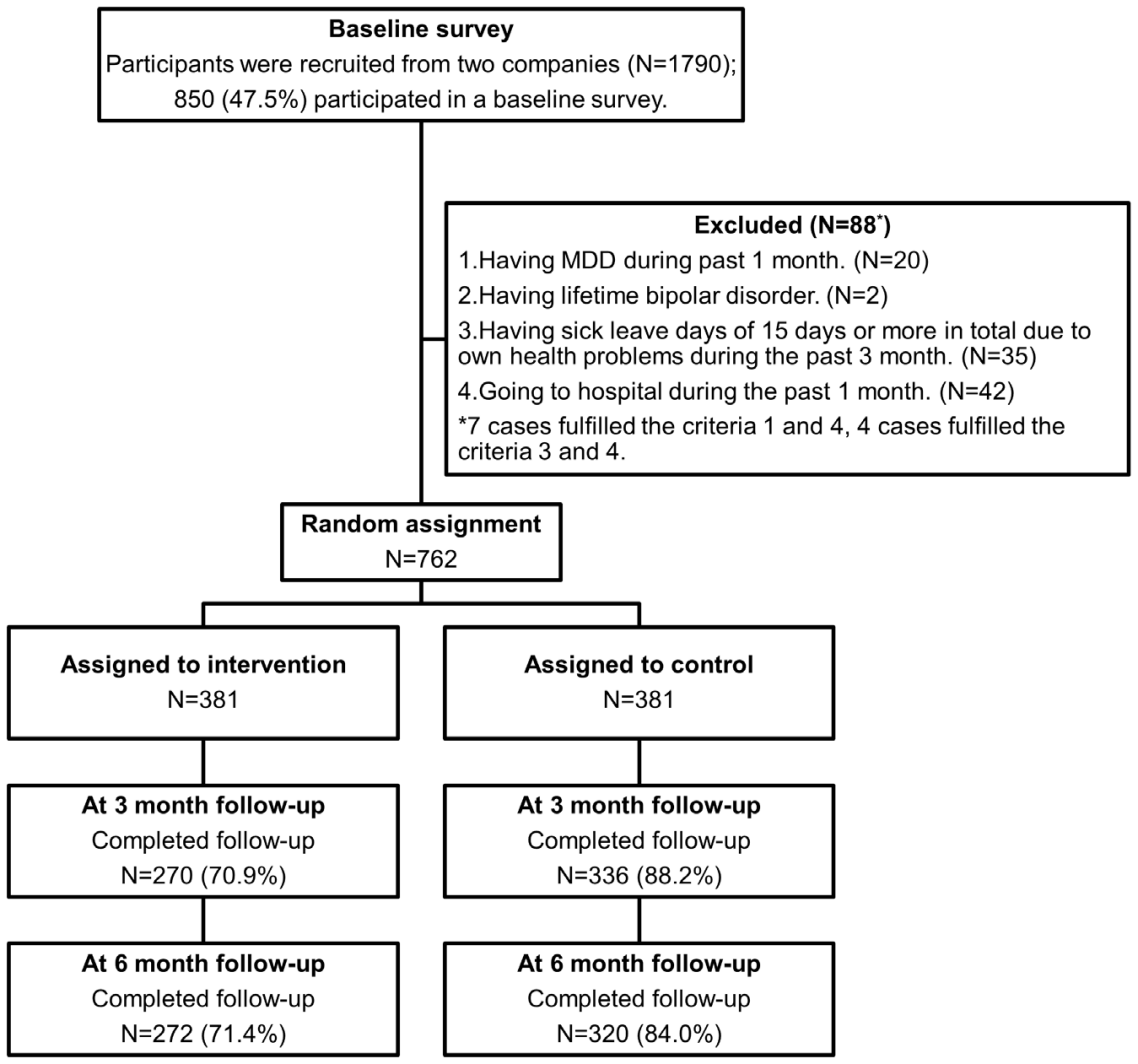
Participant flowchart.

Participant flowchart is shown in Figure 2. Participants were recruited from 2 companies (N = 1790), and 850 (47.5%) of them completed a baseline survey. Out of those, 88 had to be excluded because 20 did not fulfill criterion #1 (not diagnosed as major depressive disorder in the past 1 month), and 2 did not fulfill criterion #2 (not diagnosed as lifetime bipolar disorder), which were assessed using the web-version of WHO-CIDI 3.0. An additional 35 did not fulfill criterion #3 (not having taken 15 or more sick leave days in total due to their own health problems during the past 3 months), and 42 did not fulfill criterion #4 (not having gone to the hospital during the past 1 month). Seven of them did not fulfill criteria #1 and #4, and four of them did not fulfill criteria #3 and #4. The remaining 762 participants were randomly allocated to an intervention or control group (N = 381 for each).

At 3-month follow-up, 270 (70.9%) participants in the intervention group and 336 (88.2%) in the control group completed the follow-up survey. At the 6-month follow-up, 272 (71.4%) participants in the intervention group and 320 (84.0%) in the control group completed the follow-up survey. At each follow-up, the response rate of the control group was higher compared to that of the intervention group. Reasons for dropping out were not assessed in this study.

### Recruitment

Recruitment and the baseline survey were conducted during the period from September to October 2011. The intervention and control groups were assessed at approximately three months (from December 2011 to January 2012) and approximately six months (from March to April 2012) after the baseline survey.

### Baseline characteristics

Demographic characteristics are presented in Table 1. There were no significant differences in demographic characteristics between the intervention and control groups. In both groups, most participants were males, professionals, and university graduates with no chronic disease.

**Table 1:** Baseline characteristics of participants in the intervention and control groups.

	Intervention group (N = 381)		Control group (N = 381)		*p* *
	n (%)	Average (SD)	n (%)	Average (SD)	
**Age (years)**		38.0(9.2)		37.2(8.8)	0.24
**Gender**					0.33
Male	325(85.3)		314(82.4)		
Female	56(14.7)		67(17.6)		
**Marital status**					0.47
Never married	161(42.3)		150(39.4)		
Married	212(55.6)		226(59.3)		
Divorced or bereaved	8(2.1)		5(1.3)		
**Occupation**					0.25
Manager	93(24.4)		79(20.7)		
Professional	256(67.2)		278(73.0)		
Clerical	18(4.7)		14(3.7)		
Production	3(0.8)		3(0.8)		
Sales	3(0.8)		5(1.3)		
Others	8(2.1)		2(0.5)		
**Education**					0.59
High school	43(11.3)		34(8.9)		
Some collage	60(15.7)		70(18.4)		
University	245(64.3)		242(63.5)		
Graduate school	33(8.7)		35(9.2)		
**Chronic disease**					0.56
Yes	38(10.0)		44(11.5)		
No	343(90.0)		337(88.5)		

*t-test or χ^2^ test.

### Effects of the iCBT program on each outcome variable

Table 2 shows the means and SDs of the outcome variables at baseline, three-month, and six-month follow-ups in the intervention and control groups. Table 3 shows the estimated effects of the iCBT program on the outcome variables based on the mixed-model analyses as well as the effect sizes (Cohen's d). The iCBT program showed a significant effect on BDI-II (t = −1.99, p<0.05). The effect sizes were small. For BDI-II, the effect size was −0.14 (95% CI: −0.30 to 0.02) at the 3-month follow-up and −0.16 (95% CI: −0.32 to 0.00) at the 6-month follow-up.

**Table 2:** Means (SDs) of outcome variables at baseline, 3- and 6-month follow-up in the intervention and control groups for the whole sample.

	Intervention						Control					
	Baseline (N = 381)		3 month (N = 270)		6 month (N = 272)		Baseline (N = 381)		3 month (N = 336)		6 month (N = 320)	
	mean	(SD)	mean	(SD)	mean	(SD)	mean	(SD)	mean	(SD)	mean	(SD)
BDI-II	11.9	(8.0)	10.7	(8.6)	11.3	(9.6)	11.8	(8.0)	11.7	(8.3)	12.1	(8.7)
K6	5.6	(4.6)	5.6	(4.6)	5.7	(4.8)	5.6	(4.7)	5.8	(4.7)	6.4	(5.1)
DAS	88.0	(21.5)	87.4	(21.7)	84.9	(23.3)	87.4	(20.9)	88.5	(20.9)	87.0	(22.8)
Overtime hours during the past month	32.1	(29.5)	32.4	(28.6)	35.8	(31.1)	29.0	(25.9)	30.2	(21.8)	35.5	(27.0)
**Scores of knowledge**												
Stress management	1.6	(0.9)	2.1	(0.8)	2.2	(0.9)	1.7	(1.0)	1.8	(0.9)	1.8	(0.9)
Cognitive restructuring	1.5*	(0.9)	2.0	(0.8)	2.1	(0.9)	1.6	(0.9)	1.7	(0.9)	1.8	(0.9)
Assertive communication	1.5	(0.9)	1.9	(0.8)	1.9	(0.8)	1.6	(0.9)	1.7	(0.9)	1.7	(0.9)
Problem-solving	1.6	(0.8)	1.9	(0.8)	1.9	(0.8)	1.6	(0.9)	1.7	(0.8)	1.7	(0.9)
Relaxation training	1.5	(0.9)	1.9	(0.8)	2.0	(0.8)	1.5	(0.9)	1.6	(0.9)	1.7	(0.9)
**Scores of self-efficacy**												
Stress management	1.8	(1.0)	2.1	(0.9)	2.0	(1.0)	1.9	(1.0)	1.8	(1.0)	1.9	(1.0)
Cognitive restructuring	1.7*	(1.0)	1.9	(0.9)	1.9	(1.0)	1.7	(0.9)	1.7	(0.9)	1.7	(1.0)
Assertive communication	1.4	(0.9)	1.7	(0.8)	1.6	(0.9)	1.4	(0.9)	1.5	(0.9)	1.6	(0.9)
Problem-solving	1.7	(0.8)	1.8	(0.8)	1.8	(0.8)	1.7	(0.9)	1.7	(0.8)	1.7	(0.9)
Relaxation training	1.5	(0.9)	1.7	(0.9)	1.8	(0.9)	1.5	(0.9)	1.5	(0.9)	1.6	(1.0)

Note: BDI-II =  Beck Depression Inventory II, K6 =  Kessler's psychological distress scale, DAS =  Japanese version of the Dysfunctional Attitude Scale 24.

* The number of participants was 380 in intervention group on Knowledge of cognitive restructuring and Efficacy of cognitive restructuring at baseline.

**Table 3:** Effect of the internet-based computerized cognitive behavioral therapy (iCBT) program on primary and secondary outcome variables for the whole sample.

	Estimates of fixed effects						Cohen's d				
	Effect	SE	df	t	p	95% CI	T2-T1	95% CI	T3-T1	95% CI	
BDI-II	−0.51	0.26	621.35	−1.99	<0.05	−1.02 to −0.01	−0.14	−0.30 to 0.02	−0.16	−0.32 to 0.00	
K6	−0.29	0.17	646.59	−1.72	0.09	−0.63 to 0.04	−0.01	−0.17 to 0.15	−0.14	−0.30 to 0.02	
DAS	−1.69	0.69	625.86	−2.43	0.02	−3.05 to −0.32	−0.11	−0.27 to 0.05	−0.20	−0.36 to −0.04	
Overtime hours during the past month	−1.73	1.21	652.18	−1.43	0.15	−4.10 to 0.64	−0.04	−0.20 to 0.12	−0.13	−0.29 to 0.03	
**Improvement of knowledge**											
Stress management	0.22	0.04	650.68	5.85	<0.01	0.15 to 0.29	0.44	0.28 to 0.60	0.45	0.29 to 0.61	
Cognitive restructuring	0.20	0.04	651.23	5.17	<0.01	0.12 to 0.27	0.45	0.29 to 0.61	0.40	0.24 to 0.56	
Assertive communication	0.17	0.03	643.86	4.86	<0.01	0.10 to 0.23	0.43	0.27 to 0.59	0.40	0.24 to 0.56	
Problem-solving	0.14	0.03	635.19	4.24	<0.01	0.08 to 0.21	0.55	0.39 to 0.71	0.34	0.17 to 0.50	
Relaxation training	0.20	0.04	646.42	5.53	<0.01	0.13 to 0.27	0.46	0.29 to 0.62	0.44	0.28 to 0.60	
**Improvement of self-efficacy**											
Stress management	0.10	0.04	633.62	2.72	0.01	0.03 to 0.17	0.35	0.19 to 0.51	0.23	0.07 to 0.39	
Cognitive restructuring	0.11	0.04	638.42	2.83	<0.01	0.03 to 0.18	0.33	0.17 to 0.49	0.23	0.07 to 0.39	
Assertive communication	0.09	0.03	649.87	2.51	0.01	0.02 to 0.15	0.34	0.18 to 0.50	0.21	0.04 to 0.37	
Problem-solving	0.06	0.03	646.84	1.82	0.07	0.00 to 0.12	0.29	0.13 to 0.45	0.17	0.00 to 0.33	
Relaxation training	0.13	0.04	651.05	3.39	<0.01	0.05 to 0.21	0.32	0.16 to 0.48	0.28	0.11 to 0.44	

Note: BDI-II =  Beck Depression Inventory II, K6 =  Kessler's psychological distress scale, DAS =  Japanese version of the Dysfunctional Attitude Scale 24, T1 =  baseline, T2 =  3-month follow-up, T3 =  6-month follow-up.

#### Secondary outcomes

The iCBT program showed a significant effect on DAS (t = -2.43, p = 0.02) and all knowledge and efficacy variables (p<0.05) except for the efficacy of problem solving. The program showed a marginally statistically significant effect on K6 (t = −1.72, p = 0.09) and efficacy of problem-solving (t = 1.82, p = 0.07). Effect sizes for K6, DAS, and improvement of self-efficacy were small, while effect sizes for knowledge variables were medium.

#### NNT for improving subthreshold depression

Among respondents at the 3-month follow-up, 32.1% and 20.2% had a remission from subthreshold depression defined by BDI-II and K6, respectively. At the 6-month follow-up, 30.1% and 19.7% of respondents had a remission from subthreshold depression on BDI-II and K6, respectively. No significant difference was observed in the remission based on BDI-II between the intervention and control groups: relative risk (RR) of having subthreshold depression in the intervention group were 0.79 (p = 0.22) at the 3-month follow-up and 0.77 (p = 0.20) at the 6-month follow-up; the NNTs to achieve one remission from subthreshold depression were 14 at the 3-month follow-up and 13 at the 6-month follow-up. The probability of the remission based on K6 was not significantly different between the groups at the 3-month follow-up (RR = 1.02, p = 0.94) and was marginally significantly higher in the intervention group than in the control group at the 6-month follow-up (RR = 0.63, p = 0.06); the NNTs were -279 at the 3-month follow-up and 12 at the 6-month follow-up.

### Subgroup analyses

#### Respondents with high psychological distress

The subgroup analysis was conducted for participants who scored 5 or more on K6 at the baseline. Table 4 shows the means and SDs of outcome variables at baseline, 3-month, and 6-month follow-ups for the intervention and control groups who scored 5 or more on the K6 at the baseline (N = 194 for each group). Table 5 shows the estimated effects of the iCBT program on outcomes variables as well as the effect sizes (Cohen's d). The iCBT program showed a significant effect on BDI-II (t = −2.12, p = 0.04). The effect size for BDI-II was small, −0.16 at the 3-month follow-up and −0.25 at the 6-month follow-up.

**Table 4:** Means (SDs) of outcome variables at baseline, 3- and 6-month follow-up in the intervention and control groups: Analysis of participants with high psychological distress at baseline (K6≧5).

	Intervention						Control						
	Baseline (N = 194)		3 month (N = 125)		6 month (N = 130)		Baseline (N = 194)			3 month (N = 167)		6 month (N = 160)	
	mean	(SD)	mean	(SD)	mean	(SD)	mean	(SD)		mean	(SD)	mean	(SD)
BDI-II	16.9	(7.3)	15.4	(8.9)	15.7	(10.2)	16.3	(8.0)		15.8	(8.2)	16.2	(8.3)
K6	9.2	(3.6)	8.3	(4.6)	8.3	(5.0)	9.2	(4.7)		8.2	(4.6)	9.0	(4.7)
DAS	95.5	(19.8)	95.3	(19.8)	92.7	(22.4)	92.9	(20.9)		94.2	(19.9)	94.0	(21.1)
Overtime hours during the past month	33.9	(31.3)	33.9	(29.0)	40.8	(33.9)	28.5	(25.9)		30.3	(21.1)	35.5	(25.0)
**Scores of knowledge**													
Stress management	1.4	(0.8)	1.9	(0.7)	2.0	(0.8)	1.5	(0.9)		1.6	(0.8)	1.7	(0.8)
Cognitive restructuring	1.3*	(0.8)	1.8	(0.7)	1.8	(0.8)	1.4	(0.9)		1.5	(0.7)	1.6	(0.9)
Assertive communication	1.3	(0.8)	1.9	(0.8)	1.7	(0.8)	1.4	(0.8)		1.5	(0.8)	1.5	(0.8)
Problem-solving	1.4	(0.8)	1.9	(0.7)	1.7	(0.8)	1.4	(0.8)		1.5	(0.7)	1.6	(0.8)
Relaxation training	1.2	(0.8)	1.8	(0.8)	1.8	(0.8)	1.3	(0.8)		1.3	(0.7)	1.5	(0.8)
**Scores of self-efficacy**													
Stress management	1.5	(0.9)	1.8	(0.8)	1.7	(0.9)	1.5	(1.0)		1.5	(0.8)	1.5	(0.9)
Cognitive restructuring	1.3*	(0.9)	1.7	(0.8)	1.5	(0.9)	1.3	(0.8)		1.4	(0.8)	1.4	(0.9)
Assertive communication	1.1	(0.8)	1.5	(0.8)	1.3	(0.8)	1.3	(0.8)		1.2	(0.8)	1.3	(0.8)
Problem-solving	1.4	(0.7)	1.6	(0.7)	1.6	(0.8)	1.4	(0.8)		1.4	(0.8)	1.5	(0.8)
Relaxation training	1.1	(0.8)	1.5	(0.8)	1.5	(0.9)	1.2	(0.8)		1.3	(0.7)	1.3	(0.8)

Note: BDI-II =  Beck Depression Inventory II, K6 =  Kessler's psychological distress scale, DAS =  Japanese version of the Dysfunctional Attitude Scale 24.

* The number of participants was 193 in intervention group on Knowledge of cognitive restructuring and Efficacy of cognitive restructuring at baseline.

**Table 5:** Effect of the iCBT program on primary and secondary outcome variables: Analysis of participants with high psychological distress at baseline (K6≧5).

	Estimates of fixed effects						Cohen's d			
	Effect	SE	df	t	p	95% CI	T2–T1	95% CI	T3–T1	95% CI
BDI-II	−0.88	0.42	308.23	−2.12	0.04	−1.70 to −0.06	−0.16	−0.40 to 0.07	−0.25	−0.48 to −0.02
K6	−0.39	0.27	542.61	−1.45	0.15	−0.92 to 0.14	−0.03	−0.26 to 0.20	−0.19	−0.42 to 0.05
DAS	−2.33	0.99	295.92	−2.36	0.02	−4.28 to −0.38	−0.13	−0.36 to 0.11	−0.27	−0.51 to −0.04
Overtime hours during the past month	−1.19	1.61	345.91	−0.74	0.46	−4.35 to 1.98	−0.08	−0.32 to 0.15	−0.11	−0.34 to 0.12
**Improvement of knowledge**										
Stress management	0.18	0.05	323.55	3.66	<0.01	0.08 to 0.28	0.43	0.20 to 0.67	0.39	0.15 to 0.62
Cognitive restructuring	0.20	0.05	320.72	3.84	<0.01	0.10 to 0.30	0.63	0.39 to 0.87	0.44	0.20 to 0.67
Assertive communication	0.12	0.04	570.27	3.14	<0.01	0.05 to 0.20	0.61	0.37 to 0.84	0.29	0.05 to 0.52
Problem-solving	0.16	0.05	309.67	3.42	<0.01	0.07 to 0.25	0.71	0.47 to 0.95	0.38	0.15 to 0.62
Relaxation training	0.24	0.05	319.64	4.66	<0.01	0.14 to 0.33	0.66	0.42 to 0.89	0.53	0.29 to 0.76
**Improvement of self-efficacy**										
Stress management	0.10	0.05	310.38	2.09	0.04	0.01 to 0.20	0.39	0.16 to 0.63	0.24	0.01 to 0.48
Cognitive restructuring	0.09	0.05	312.70	1.66	0.10	−0.02 to 0.19	0.41	0.18 to 0.65	0.21	−0.03 to 0.44
Assertive communication	0.14	0.05	320.71	2.96	<0.01	0.05 to 0.23	0.57	0.33 to 0.81	0.34	0.10 to 0.57
Problem-solving	0.07	0.05	315.91	1.60	0.11	−0.02 to 0.16	0.38	0.15 to 0.62	0.20	−0.03 to 0.43
Relaxation training	0.16	0.04	622.71	3.71	<0.01	0.07 to 0.24	0.40	0.17 to 0.64	0.34	0.10 to 0.57

Note: BDI-II =  Beck Depression Inventory II, K6 =  Kessler's psychological distress scale, DAS =  Japanese version of the Dysfunctional Attitude Scale 24, T1 =  baseline, T2 =  3-month follow-up, T3 =  6-month follow-up.

The iCBT program showed a significant effect on DAS (t = −2.36, p = 0.02), all knowledge variables, efficacy of stress management, assertive communication, and relaxation training (p<0.05). While the effect sizes of DAS and self-efficacy were small, those for variables on knowledge were medium.

#### Process evaluation

Table 6 shows the process evaluation indicators of the iCBT programs only for the intervention group. Most (89%) participants in the intervention group completed Lesson 1, and 65% submitted their homework after completing this lesson. The proportion of those who completed lessons and submitted homework gradually decreased during the later lessons. About two thirds of the intervention group completed all six lessons, while only a quarter of them submitted all six homework assignments. The average number of lessons that the respondents received was 4.5. The average number of homework assignments submitted was 2.7. Three quarters of the intervention group completed at least three lessons, and about half (45%) submitted at least three homework assignments.

**Table 6:** Progress of learning in the iCBT program among the intervention group participants: the whole sample.

Contents	Completers of lessons	Submitters of homework
Lesson 1 *Learning about stress*	340(89.2%)	248(65.1%)
Lesson 2 *Knack for self case formulation based on CB model*	317(83.2%)	193(50.7%)
Lesson 3 *Try cognitive restructuring part 1*	291(76.4%)	176(46.2%)
Lesson 4 *Try cognitive restructuring part 2*	272(71.4%)	149(39.1%)
Lesson 5 *Knack for communication*	258(67.7%)	127(33.3%)
Lesson 6 *How to solve your problem effectively*	247(64.8%)	117(30.7%)
All six lessons	247(64.8%)	93(24.4%)
Average number of respondents who completed per lesson (proportion in to the total sample, %).	287.5(75.5%)	
Average number of respondents who submitted homework per lesson (proportion to the total sample, %).	168.3(44.2%)	
Average number of sessions completed per respondent.	4.53	
Average number of homework submitted per respondent.	2.65	
The number of respondents who completed more than 3 lessons (proportion to the total sample).	291(76.4%)	
The number of respondents who submitted homework more than 3 times (proportion to the total sample).	173(45.4%)	

## Discussion

The present RCT examined the effects of a newly developed iCBT program for improving the symptoms of depression and other outcomes at three- and six-month follow-ups among workers employed in private companies in Japan. In the total sample, the iCBT program showed a significant intervention effect on depression, as measured by BDI-II, with the effect sizes of −0.16 at the six-month follow-up. The iCBT program also showed a marginally statistically significant intervention effect on psychological distress measured by K6 and significant intervention effects on improving dysfunctional attitude, knowledge, and self-efficacy of most components of CBT. This iCBT program may effectively improve depressive symptoms among workers with the universal approach (i.e., targeting the whole working population), even though the effect size may be modest.

To our knowledge, the present study first demonstrated that an Internet-based computerized CBT was effective in improving depression in a non-clinical working population. In the intervention group, BDI-II scores improved at the three-month follow-up and returned to the baseline level at the six-month follow-up. In the control group, BDI-II scores stayed at almost the same level at the three-month follow-up and increased slightly by the six-month follow-up. This difference yielded effect sizes of −0.14 and −0.16 on BDI-II scores at the three- and six-month follow-ups, respectively. After the baseline survey, one of the two companies that participated in the study merged with another company. The observed increase in depression in the control group was partly attributable to the increased workload associated with the organizational change. The current iCBT program seems to improve depression to some extent under severe working conditions. With consideration for the fact that an iCBT can be delivered to many workers at a low cost, this iCBT may be a promisingly cost-effective approach for improving depression among workers. The effect size of the current iCBT program for improving depression was small (around 0.15). Grime (2004) reported that the effect sizes (Cohen's d) of CCBT on depression among workers who recently had stress-related absenteeism were −0.25 (p = 0.04) at one month after the end of intervention and −0.1 (p = 0.56) at the three-month follow-up [18]. The current iCBT program might be effective in improving depression among workers to a similar or greater extent compared to the ordinary CCBT program. The small effect sizes in the present study could be attributable to the non-clinical sample and the lower degree of therapist involvement in this iCBT program [16], [17]. In fact, the iCBT program showed a better intervention effect on BDI-II, when the analysis was limited to those who have high depression score at baseline (−0.16 and −0.25 at the three- and six-month follow-ups, respectively), which is concordant with a previous meta-analysis that the effect of CBT programs in preventing major depressive disorder was greater for selective and indicated interventions than the universal one [33].

In the present study, the NNTs to achieve one remission from subthreshold depression were 14 at the 3-month follow-up and 13 at the 6-month follow-up for BDI-II. These were −279 at the 3-month follow-up and 12 at the 6-month follow-up for K6. These figures were larger than those of the previously reported NNTs of iCBT for improving depression among patients with major depressive disorder (2 to 4), when compared with TAU or waiting list [32]. However, these were lower (thus better) than a NNT that reported a RCT using the automated e-mails containing general information and advice about self-help strategies for depression to achieve one remission from subthreshold depression (26) [38]. The NNTs of the present iCBT program fall between these studies of clinical and non-clinical populations.

The iCBT program showed a significant intervention effect on dysfunctional attitude measured by the DAS in terms of knowledge and efficacy of most CBT components except for self-efficacy of problem solving. Dysfunctional attitude is one of major targets of cognitive restructuring, a component of the current program. The present findings are consistent with previous ones that a CCBT program was effective in improving dysfunctional attitudes among medication-free participants with major depressive disorder [39], while the present study observed a larger effect size than that of the previous study (0.5). The effect sizes for knowledge were better than the one reported by a previous worksite-based study [40]. In addition, improved levels of knowledge and self-efficacy were maintained at the six-month follow-up. Downloadable PDF summaries of sessions of the iCBT program may have helped participants maintain their knowledge and self-efficacy. This may also be a benefit of using Manga story as a part of the iCBT program, while we did not systematically assess it.

The process evaluation of the iCBT program indicated that proportions of those who completed lessons and submitted homework decreased for the later lessons. About two-thirds of the intervention group completed all six lessons, while only a quarter of them submitted all six homework assignments. The completion rates of self-help programs without clinician contact were generally low [13]. The average number of completed lessons per respondent (4.5) in this six-lesson iCBT program was very close to that of a previous seven-chapter self-help iCBT program [41], [42]. The low rates of completing the lessons and submitting homework may have resulted in a smaller effect size in this study. It would be critical to increase completion rates to improve the effect of an iCBT program.

### Limitations

This study has several limitations. First, participants were recruited from two IT companies in Japan. Most participants were males, working as professionals, and university graduates. They had their own PCs in their offices or homes. The participants were also supposed to have experience using a PC and studying through online programs. Higher education level may also help participants learn from the iCBT program. The generalization of the present findings to the general working population is limited. Second, the rate of completing lessons and homework was low. Only 93 participants submitted all homework assignments, and the average number of homework assignments submitted was 2.7 per respondent. This may weaken the findings. Third, the dropout rates in the present study were 29.1% and 28.6% at the three- and six-month follow-ups, respectively. In their systematic review, Kaltenthaler et al. (2008) reported that average CCBT dropout rates were 18–35% [43]. Wantland et al. (2004) reported that an average dropout rate was 21% [44]. The dropout rates in the present study were within the range. However, the dropouts may cause a selection bias, particularly if the intervention group participants with higher levels of depression were more likely to quit the program. Fourth, the present study provided the participants in the control group with e-mails providing stress management tips. This may weaken the intervention effect. Fifth, there was the possibility that participants in the control group could have information about the iCBT program from participants in the intervention group at same workplace. This contamination may weaken the intervention effect. Sixth, all outcomes in the present study were measured by self-report, which may be affected by the perception of participants or situational factors at work. A further RCT should be conducted to examine whether the iCBT program is effective in a larger sample of workers with diverse characteristics, particularly in terms of occupation and education.

## Supporting Information

Checklist S1 CONSORT 2010 checklist of information to include when reporting a randomized trial.
(DOCX)

Protocol S1 The original protocol in Japanese.
(DOCX)

Protocol S2 English translation of the original Japanese protocol.
(DOCX)
