# Supplementary material for: Effects of an Internet-Based Cognitive Behavioral Therapy (iCBT) Program in Manga Format on Improving Subthreshold Depressive Symptoms among Healthy Workers: A Randomized Controlled Trial
Source: PLoS One. 2014 May 20;9(5):e97167. doi: 10.1371/journal.pone.0097167 (PMC4028193; doi:10.1371/journal.pone.0097167)
Supplement: Protocol S1 — The original protocol in Japanese. (DOCX) [file pone.0097167.s002.docx]

労働者に対するインターネット認知行動療法プログラムの効果：無作為化比較試験

（略称「労働者向けiCBT効果評価」）

川上憲人、今村幸太郎、古川壽亮、松山　裕、島津明人、馬ノ段梨乃、川上苑子、上田芙基子

本臨床試験はUMIN 臨床試験登録 (UMIN-CTR) (登録番号: UMINxxxxxxxxx)に登録予定である。

# 1. 背景

うつ病は最も高頻度にみられる精神疾患の１つで、世界中で３億4000万人が罹患しており、生活の質の大きな低下や、地域や職場での経済的損失と関連している([Hosman, Jané Llopis, & Saxena, 2004](#_ENREF_15); [Kessler et al., 2006](#_ENREF_19); [Saarni et al., 2007](#_ENREF_29))。故に、うつ病の第一次予防は世界精神保健において重要な戦略である。

認知行動療法 (cognitive behavioral therapy; CBT)([Aaron T. Beck, 1967](#_ENREF_3), [1979](#_ENREF_4))に基づく介入プログラムが労働者のうつ病や不安の改善に効果があることを示す文献が増えている。メタ分析によると、CBTの効果の推定値はCohen’s d = 0.68であった([van der Klink, Blonk, Schene, & van Dijk, 2001](#_ENREF_36))。他のメタ分析では、CBTの効果の推定値はCohen’s d = 1.16であった([Richardson & Rothstein, 2008](#_ENREF_26))。さらに、CBTはうつ病の予防にも効果的は可能性がある。２つのメタ分析は、CBTが大うつ病性障害の予防に効果があることを示している([Cuijpers, Munoz, Clarke, & Lewinsohn, 2009](#_ENREF_7); [Cuijpers, van Straten, Smit, Mihalopoulos, & Beekman, 2008](#_ENREF_9))。しかしながら、これらのプログラムにはCBTについて十分な訓練を受けた専門家が必要となる理由から、これらの介入の推進には未だ限界がある([World Health Organization. Department of Mental Health and Substance Abuse., 2001](#_ENREF_37), [2005](#_ENREF_38))。加えて、もしサービスが提供できたとしても、時間、費用、メンタルヘルス介入に関連する偏見が、効果的な介入へのアクセスへの障害として機能する可能性がある([Ruwaard, Lange, Bouwman, Broeksteeg, & Schrieken, 2007](#_ENREF_28))。

CBTに基づく介入を広く提供するための革新的な方法として、コンピューターによるCBT (computerized CBT; CCBT) と、CCBTをインターネットで提供する方法 (iCBT) がある。CCBTとiCBTは、対面CBTと同様の原則に基づく基礎的な情報と技術を、教育的レッスン、ホームワーク、補助的資源を含む、高度に構造化されたフォーマットで教える([Titov, Andrews, & Sachdev, 2010](#_ENREF_35))。先行研究は、臨床条件におけるCCBTとiCBTによるうつ病や不安の有意な改善効果を示している([Andrews, Cuijpers, Craske, McEvoy, & Titov, 2010](#_ENREF_2))。iCBTプログラムは、高い匿名性とアクセス性から特に有益である([Gega, Marks, & Mataix-Cols, 2004](#_ENREF_12); [Spek et al., 2007](#_ENREF_33))。

しかしながら、特に一般労働人口を対象としたiCBTプログラムは無く、無作為化比較試験 (randomized controlled trial; RCT) を用いた効果研究も無い。加えて、DSM-IV([American Psychiatric Association., 2000](#_ENREF_1))などの標準化された基準により診断されたつ病のリスク低減についてのiCBTの有益な効果をRCTにより検討した先行研究は無い。

この無作為化比較試験では、以下を明らかにすることを目的とする。

1. 日本において、閾値下抑うつ症状をもつ労働者向けの６週間のiCBTプログラムを新たに開発する
2. 日本において、閾値下抑うつ症状を持つ労働者に対するiCBTプログラムの、３、６ヶ月後追跡調査時点での抑うつ症状の改善効果を検討する。
3. 日本人労働者において、DSM-IVによる大うつ病エピソード (major depressive episode; MDE) の、６ヶ月後追跡調査時点でのリスクを低減させる。
4. 日本人労働者に対するワーク・エンゲイジメントおよび仕事の生産性の改善効果を検討する。

本研究は、TAUをコントロールとした2群並行群間比較試験であり、非盲検化無作為割り付け臨床試験である。

# 2. 目的

## 2.1 主たる臨床的疑問

主たる臨床的疑問は以下のとおりである：

“閾値下の抑うつ(subthreshold depression)を呈する労働者において、標準的な従業員支援プログラム(employee assistance program; EAP)に、認知再構成、アサーティブネストレーニング、問題解決技法からなるインターネットCBTプログラムを提供することで、企業内EAPのみの場合よりも、プログラム終了後における抑うつの重症度（BDI-IIにより測定）が低く、また６ヶ月の追跡期間における大うつ病エピソード（WHO-CIDIウェブ版で測定）が減少するかどうか？”

本研究におけるプライマリ・アウトカムは以下のとおりである：

1. 無作為割り付けから3, 6ヶ月後の抑うつ重症度(BDI-IIにより測定)
2. 無作為割り付けから6ヶ月間の大うつ病エピソードを発症するまでの期間(WHO-CIDIウェブ版により測定)

これら2つのプライマリ・アウトカムは、iCBTによる介入の効果を判断するために用いられる。

## 2.2. 第2の臨床的疑問

セカンダリ・アウトカムには以下が含まれる：

無作為割り付けから3,6ヶ月時点での

1. 抑うつ重症度(Kessler’s psychological distress scale; K6)
2. 労働パフォーマンス
   - 1. 過去1ヶ月の労働生産性(WHO Health and Work Performance Questionnaire; HPQ)
     2. 過去3ヶ月間の疾病休業日数
3. ワーク・エンゲイジメント（Utrecht Work Engagement Scale; UWES）
4. 非機能的態度（24-item Dysfunctional Attitude Scale; DAS-24）
5. iCBTプログラムの５つの構成要素（ストレス対処、認知再構成、アサーティブなコミュニケーション、問題解決、リラクセーション）における知識と自己効力感の改善
   1. 知識：「～について、あなた自身がどのくらいご存知か教えてください」
   2. 自己効力感：「～について、自分でどのくらいできていると思いますか」

6) iCBTプログラムを受けた参加者の介入終了時点での満足度と詳細な事後アンケート

## 2.3. サブグループ解析

プログラムの効果は、抑うつの当初の重症度によって異なると考えられる。無作為割り付け前の抑うつの高低（K6が５点以上/４点以下）を層別化要因のひとつとして扱い、K6が５点以上のグループでサブグループ解析を行う(selective intervention effect)。また、K6が４点以下のグループでも同様の解析を行う。加えて、全対象者でも解析を行う(universal intervention)。

## 2.4. 有害事象

本研究の介入から、健康への有害事象は想定されないが、抑うつの重症化(究極的には自殺)の可能性のみが存在する。深刻な希死念慮等が生じた場合を想定し、緊急用の電子メール、電話番号を事務局に用意する。臨床研究コーディネーター(CRC, 今村幸太郎, MS, MPH)は臨床心理士で、緊急メールないし電話に最初に対応し、スーパーバイザー(川上憲人, MD, PhD)に、適切な対応方法について相談する。

抑うつの重症化はプライマリ・アウトカムならびにセカンダリ・アウトカムで測定する。自殺未遂、自殺の完遂および入院については、6ヶ月フォローアップにて測定する。本研究では、ストッピング・ルールを設けない。なぜなら、有害事象が本研究への参加と明らかに関連したものかどうか、また、すべてのサンプルの登録を完了する前に、アウトカム・データが主たる臨床的疑問に答えるに充分なものかどうかを明らかにするだけの充分大きなサンプルを扱わないためである。

研究責任者である川上は、すべての決定について責任を負う。川上は、報告された有害事象と自殺の危険性があるすべてのケースについて、24時間以内に確認する。出張やその他の業務のためにこの役割を果たせないときには、その他の任命された臨床研究者が参加者の安全を確認する。

# 3. 参加者

## 3.1. 研究母集団

閾値下抑うつを呈する労働者男女が以下の基準にしたがって抽出される：

### 3.1.1. 包含基準

1. エントリー時20～60歳
2. 男性および女性
3. 現在フルタイムで雇用されている正規社員
4. スクリーニング時にK6スコアが5点以上 （K6が４点以下の者も含めた全対象者によるサブ解析も行う）
5. 自宅または職場のPCからインターネットにアクセスできる者

### 3.1.2. 除外基準

1. 非正規、パートタイム職員
2. 過去3ヶ月に健康上の理由による病欠が15日以上ある
3. 過去1ヶ月間にメンタルヘルスの問題について専門家からなんらかの治療を受けた人
4. 過去1ヶ月間に大うつ病エピソードが存在する(WHO-CIDIウェブ版により確認)
5. 双極性障害の既往がある(WHO-CIDIウェブ版により確認)

２つの調査サイト（ある企業Aのすべての正規社員290人、および全社で約11,000人規模の別企業Bの３つの部門の正規社員約1500人)を登録し、参加者募集の電子メールを送付する。

## 3.2. サンプルサイズの前提と推定

閾値下抑うつに対する心理学的な治療(その多くはCBTであるが)の系統的レビューでは、介入終了後でCohenの dが 0.42 (95%CI: 0.23 - 0.60)という数値を導いている([Cuijpers, Smit, & van Straten, 2007](#_ENREF_8))。Cohenの d が .42というのは、治療効果(アウトカムの測定値における対照群の平均と介入群の平均の差)が、0.42標準偏差分であることを示す。

| Alpha | power | Effect size | N in each cell  (The allocation ratio = 1:1) | Total N |
| --- | --- | --- | --- | --- |
| 0.05 | 0.90 | 0.4 | 131 | 262 |
| 0.05 | 0.80 | 0.4 | 98 | 196 |

したがって、介入群に131名を、対照群に同数、合計262名を無作為割り付けすると、標準偏差0.40に相当する群間差を想定した治療効果を、90%の検出力で検出することになる。しかしながら、これらの計算は脱落を考慮していない。全体の75%が6ヶ月フォローアップで回答すると考えると、6ヶ月後には介入群が98名、対照群が98名となる。この場合は、90%の検出力で0.47SDに相当するエフェクト・サイズを同定することになり、本臨床試験における6ヶ月の時点で期待される効果の最良の推定値と等しいものとなる。

## 3.3. 参加者のリクルート

企業代表者および(または)研究責任者は各事業所を訪れ、可能であれば小グループ単位で労働者に会う。そして、本研究の目的と手続きの概要を説明し、あらかじめ本プログラムへの関心を高めるようにする。

CRCは、1700名の被雇用者に対して、参加者募集のメールを送付する。このうち、680名が調査への参加に同意すると予想され、うち272名が抑うつありと判定されると考えられる(K6>=5)。このうち245名が適格になると考えられる。この245名が積極的な介入群(n=122)と待機群(n=123)に無作為割り付けされる。この後、労働者に対してiCBTプログラム（６週間）を提供する。待機群は無作為割り付けから6ヶ月以降にiCBTを受けることができる。

### 3.3.1. 適格性のアセスメント

参加者募集のメールを、対象となる部署のすべての労働者に送付する。適格性のアセスメントはベースライン調査時に行われる。研究に関するすべての説明を記載したウェブサイトを用意する。労働者は、ウェブサイトにアクセスし、調査の説明書を読み、調査参加同意のボタンをクリックして、ベースライン調査ページに進む。

現在大うつ病エピソードにある労働者は、臨床試験からは除外されるが、CRCがコンタクトをとり、産業医ないしは外部の専門家を受診するよう助言する。CRCはそれまでに得られたすべてのデータに基づき、iCBTよりも専門家に直接受診した方が利益が大きいと判断されたことをメールにより説明する。

### 3.3.2. ベースライン・アセスメント

ベースライン・アセスメントは、第1回調査を兼ねる。したがって、ベースライン・アセスメントには、以下の変数が含まれる。

1. 性別
2. 年齢
3. 職種、学歴、婚姻状態
4. 過去1ヶ月についてのK6
5. 過去2週間についてのBDI2
6. ワーク・エンゲイジメント
7. Health and Productivity Questionnaire
   - 過去4週間の労働時間、過去4週間の絶対的および相対的absenteeism、過去4週間の絶対的および相対的presenteeism
8. 身体的疾患の治療中か否か、精神的疾患の治療中か否か
9. 過去3ヶ月の病欠日数
10. 気分障害に関するCIDIの結果

### 3.3.3. 説明と同意

本研究の目的と手続きについて開示・説明がなされた後に、すべての参加者から調査参加同意のボタンをクリックして、第一回質問紙に回答することで同意を得る。

候補者には、参加は任意であること、参加後においても理由を告げずに介入およびアセスメントを辞退することができること、本研究への参加・不参加は会社における利益・不利益につながらないことを説明する。各参加者のデータは、機密性を保つために、順に割り振られた番号で扱われ、インターネットに接続しないコンピューターに保管され、CRCのみがアクセス可能となる。

### 3.3.4. 介入への割り付け

適格性のある対象者を介入群と対照群とに1:1の割合で割り付けする。無作為割り付けは2つの変数について層別化する：ベースラインにおける抑うつ重症度(K6=<4 vs. >=5)、事業所(会社A、会社B)。対象者が２箇所からリクルートされると仮定し、合計４の層となる。本研究における統計学者は、事前に４の無作為割り付けリストを用意し、４層のうちのいずれかひとつを用いる。各リストは、ひとつの層あたり介入群対対照群の比が1:1になるようにブロック化される。統計学者は、本研究の他の業務を担当しないリサーチ・アシスタントにこのリストを送り、リサーチ・アシスタントは安全な場所でこのリストを管理し、割り付けの隠蔽化が可能なスプレッド・シートを作成する。

無作為割り付けの前に、CRCはその人が無作為化対照試験における適格性を有するかどうかの最終確認を行う。たとえば、無作為割り付けの前に、過去１ヶ月間の気分障害の既往がある者を除外する。CRCは適格性を有する参加者のリストを作成し、各参加者がどの層に該当するかを確認し、上記スプレッド・シートに入力する。この入力はいったん行うと変更不可能である。

対照群に割り付けられた人は待機リストに載り、希望があれば割り付けから6ヶ月以降にiCBTを受けることができる。

ひとつの層別化基準はK6>=5 と K6<=4である。このカットオフは、先行研究において地域住民と気分障害や不安障害をもつ患者とを分けるK6の最適カットオフ値が4/5であるため選択された([Sakurai, Nishi, Kondo, Yanagida, & Kawakami, 2011](#_ENREF_30))。

# 4. 介入

## 4.1. 手順とスケジュール

無作為割り付けと介入の開始との時間間隔が可能な限り短くなるよう、調査参加登録締め切り後、ただちにCRCは割り付けを行い、できるだけ早くにiCBTプログラム受講の案内メールを送付するよう努力する。

介入群に割り付けられた参加者は、『仕事に役立つメンタルヘルスシリーズ』という新たに作成されたiCBTプログラムを学習する。このプログラムはストレス対処のスキルを提供する６週間のウェブトレーニングコースである。このプログラムは全６回で構成され、毎週１回ずつ提供される。１回分の学習時間は宿題を含めて約30分である。このプログラムはインターネット環境があればどこでも利用できる。

本プログラムの特徴の１つは、学習参加者の理解を促進するために、臨床心理士と相談にきた従業員の漫画ストーリーに沿ってトレーニングを提供することである。先行研究を通して、漫画のストーリーやキャラクターを教育に用いるいくつかのメリットが知られている。一つは、それらの素材が個人を動機付ける助けとなる点で、高い動機づけはプログラムへの参加継続に役立つ([Hutchinson, 1949](#_ENREF_16))。二つ目は、学習しやすさを促進する点である。テキストに漫画ストーリーを組み合わせたプログラムは文章のみのプログラムと比較して学習者がより容易に内容を理解できる([Hutchinson, 1949](#_ENREF_16); [Sones, 1944](#_ENREF_32))。三つ目に、漫画ストーリーは学習者のプログラムへの関心を大きくする([Sones, 1944](#_ENREF_32))。多くの日本人労働者は漫画に親しんでおり、これらのメリットは職場での教育においても応用できる。

本iCBTプログラムには、セルフモニタリング、認知再構成、アサーティブネス、問題解決、リラクセーションのスキルが含まれる。各レッスンの最後には、参加者は任意ではあるが、学習内容の理解を促進するための宿題の提出を求められる。宿題を提出した参加者には訓練されたスタッフ（臨床心理士）からのコメントがフィードバックされる。

参加者はベースライン調査から10週間で全６回の学習を完了する。未学習の参加者には、研究事務局から毎週月曜にリマインダメールが送信される。

企業内EAPプログラムは、既にこの企業において提供されている。プログラムには、すべての社員が利用可能な精神的健康・身体的健康に関するホームページ、必要に応じて電話カウンセリングないし対面カウンセリングのサービス、管理職および社員に対するメンタルヘルスに関する講義が含まれている。

　対照群の参加者には、月１回、「ストレス解消に役立つ豆知識」と題したメールマガジンも提供する。メールマガジンは各回500字程度で、ストレス対処のヒントが含まれる。内容は次の通りである。1)ストレス解消と睡眠、2)ストレス解消と食事、アルコール、3)ストレス解消と休日の過ごし方、4)ストレス解消と音楽、5)入浴ストレス解消とエクササイズ。

## 4.2. カウンセラーのアドヒアランスの測度(クオリティ・コントロール)

本研究で使用されるiCBTプログラムは、研究者によって認知行動理論に基づき開発された。厳密に同一の内容が、東京大学精神保健学分野に設置されたサーバーのウェブ学習管理システムにより、各セッションで介入群の全参加者に提供される予定である。

参加者が提出した宿題への個別のフィードバックコメントについては、宿題は５人の修士、博士、ポスドクレベルの心理士により評価されコメントされる。フィードバックコメントの質を同様に保つために、これらの心理士には簡単な指示と半日のトレーニングが、例を含んだマニュアルとともに熟練した臨床心理士である、研究者の１人(今村幸太郎)によって提供される。宿題へのすべてのフィードバックコメントは記録され、研究者の１人(今村)が評価することで、これらの心理士の実績をモニターし、必要に応じて追加のトレーニングを提供する。

## 4.3. クライエントのアドヒアランスの測度

CRCはクライエントのアドヒアランスを確認し、以下のいずれかに該当する場合にはメールによりリマインダを送付する：

1. 学習開始を依頼するメールから2週間以上、学習していない。
2. セッション間が2週以上、連絡なく経過している

# 5. アセスメント

## 5.1. 測度

### Beck Depression Inventory-II (BDI-II)

ベック抑うつ尺度のオリジナルは1961年に公刊されており([A. T. Beck, Ward, Mendelson, Mock, & Erbaugh, 1961](#_ENREF_6)), 抑うつ重症度の自己評価尺度として広く用いられている。DSM-IVの使用開始により質問項目の時間的枠組みがBDI第2版で改訂され、その信頼性・妥当性が確認されている([Aaron T. Beck, Steer, & Brown, 1996](#_ENREF_5))。日本版の信頼性と妥当性は高いことが見出されている([Hiroe et al., 2005](#_ENREF_14))。

ベースライン、3ヶ月後(介入終了後)、6ヶ月フォローアップにてBDI-IIを施行する。

### Composite International Diagnostic Interview (CIDI)

CIDIは精神障害を査定するために広く用いられている構造化診断面接で、訓練を受けた一般の面接者が一般母集団に施行するものとなっている([Robins et al., 1988](#_ENREF_27))。DSM-IV に合わせて改訂がなされている。我々は最新のコンピューター化された版を用い([Kessler & Ustun, 2004](#_ENREF_24))、気分障害およびアルコール関連のセクションを実施する。CIDIと標準化された臨床的アセスメントとの一致は既に報告されている([Haro et al., 2006](#_ENREF_13))。HPQとCIDIの日本語版は日本におけるWorld Mental Health Survey で使用されている([Kawakami et al., 2005](#_ENREF_17))。

本研究ではCIDIをweb化し参加者が自ら回答する形式をとる([Kawakami et al., 2008](#_ENREF_18))。

### Kessler’s psychological distress scale (K6)

K6は近年開発された非常に短い(6項目)自己報告式質問紙で、よく見られる精神障害のスクリーニングのためのものである([Kessler et al., 2002](#_ENREF_21))。 これは項目反応理論に基づくもので、一般母集団の分布の90～99パーセンタイルで、回答者の弁別力を最大にする項目から構成されている。なぜなら、どのような時点においても、5～10%の人々が精神障害を有することが知られているためである。様々な社会・人口統計学的な特徴において心理測定特性が安定して示されている項目のみが最終的に含まれている。K6はこれまでの広く用いられてきたスクリーニング質問紙と同等あるいはそれ以上に良いものであることが見出されている([Furukawa, Kessler, Slade, & Andrews, 2003](#_ENREF_11); [Kessler, Barker, et al., 2003](#_ENREF_23))。日本語版の妥当性は既に示されている([Furukawa et al., 2008](#_ENREF_10))。

本研究ではK6を最初のスクリーニングとして用いる。スクリーニングに加えて、3ヶ月後、6ヶ月フォローアップ(これらすべて統制群にも実施)で、セカンダリ・アウトカムの測度として扱われる。

### Health and Productivity Questionnaire (HPQ)

世界保健機構のHealth and Productivity Questionnaire (HPQ) は、健康問題による職場のコストを推定するために開発された自己報告式尺度で、自己報告による病欠(absenteeism)と仕事のパフォーマンスの低下(presenteeism)から構成されている。妥当性検証研究では、HPQで測定された労働時間と勤務記録との有意な相関(r=0.61 ～0.87)([Kessler, Barber, et al., 2003](#_ENREF_22))、管理者による仕事の生産性評価との有意な相関(r=0.52)([Kessler et al., 2004](#_ENREF_20))、その他の事務記録との有意な相関(曲線下面積、0.58-0.72)があることを見出している([Kessler & Ustun, 2004](#_ENREF_24))。

ベースライン、3ヶ月後(介入終了後)、6ヶ月フォローアップにてHPQを施行する。

### Sick leave days during past 3 month

参加者は、過去３ヶ月間の疾病休業日数について報告を求められる。

ベースライン、3ヶ月後(介入終了後)、6ヶ月フォローアップにて、過去３ヶ月間の疾病休業日数を質問する。

### Utrecht Work Engagement Scale (UWES)

ワーク・エンゲイジメントは日本語版Utrecht Work Engagement Scale (UWES)によって査定される([Shimazu et al., 2008](#_ENREF_31))。UWESは３つの下位尺度（活力、熱意、没頭）からなる全９項目の尺度である。項目は７件法（０～６）で測定される。項目例としては、「仕事をしていると，活力がみなぎるように感じる。」、「仕事に熱心である。」、「私は仕事にのめり込んでいる。」などがある。合計値は９項目の和で計算される。

ベースライン、3ヶ月後(介入終了後)、6ヶ月フォローアップにてUWESを施行する。

### The 24-item Dysfunctional Attitude Scale (DAS-24)

24項目版非機能的態度尺度(DAS-24)は、非機能的態度尺度の短縮版であり、抑うつスキーマを測定する自己記入式の尺度である([Power et al., 1994](#_ENREF_25))。それぞれの項目は７件法（１～７）で、高得点ほど非機能的態度が強いと評定される。日本語版の信頼性・妥当性は既に検証済みである([Tajima et al., 2007](#_ENREF_34))。

ベースライン、3ヶ月後(介入終了後)、6ヶ月フォローアップにてDAS-24を施行する。

### Improvement of knowledge and self-efficacy

参加者は、iCBTの５つの構成要素（ストレス対処、認知再構成、アサーティブコミュニケーション、問題解決、リラクセーション訓練）に関する知識と自己効力感の改善について評定することを求められる。知識の改善は「～について、あなた自身がどのくらいご存知か教えてください」という質問により査定され、自己効力感の改善は「～について、自分でどのくらいできていると思いますか」という質問により査定される。どちらも５件法（０～４）で評定される。・

ベースライン、3ヶ月後(介入終了後)、6ヶ月フォローアップにて、知識と自己効力感の改善について質問する。

## 5.2. フォローアップの手順とスケジュール

### 5.2.1. 介入からの脱落

参加者はいつでも介入および(または)研究への参加を辞退することができる。もし介入への参加を中断するとしても、3,6ヶ月後のフォローアップでアウトカム・データを収集するあらゆる努力をしなければならない。

新たな精神症状が生じたり、精神症状の増悪が生じた場合には、iCBTの実施中であってもそうでなくても、すべての参加者は企業から提供されているEAPサービスを通常どおり使用したり、企業外部の専門的援助を求めることができる。さらに、研究センターの心理士につながる電子メールあるいはホットラインに電話をかけることができる。

本プログラム以外の治療が望ましいと考えられるような、新たな精神症状の発生、現存する閾値下抑うつの増悪等がiCBT中に見られた場合には、カウンセラーは参加者に対して、産業医、かかりつけ医、ないしメンタルヘルスの専門家に直接相談することを勧めなければならない。参加者が、iCBTと新たに勧められたその他の治療との間に葛藤を感じなければ、介入への参加は継続され、もしなんらかの葛藤を感じたり、参加者の側から介入の辞退の要望があった場合には中止しなければならない。iCBTが中止となった場合でも、参加者は3,6ヶ月後のフォローアップには参加するよう促される。

### 5.2.2. 研究からの脱落

3ヶ月後および6ヶ月後のフォローアップに対して同意を撤回した参加者は、研究からの脱落とみなされる。

### 5.2.3. どのようにアドヒアランスを高め、脱落を減らすか

介入からの脱落は研究からの脱落とは厳密に区別される。クライエントには。iCBTを中止したとしても、可能な限り最終的なアセスメントへの回答を依頼する。

　iCBTの学習、3,6ヶ月後の調査においては、無回答者には少なくとも２回以上、メールで参加を促す。

## 5.3. 反応測度のアセスメント

### 5.3.1. 自己報告式質問紙の独立性

本研究では、すべてのアウトカム・データはweb上のアンケートで収集される。評価の匿名性と独立性を保つために、CRCのみが収集されたデータにアクセスできることとする。

### 5.3.2. データ収集

3ヶ月フォローアップ (介入直後のアウトカム)

介入群においては、プライマリ・アウトカムは無作為割付けから3ヶ月後(iCBT学習期間終了から２週間後)に測定される。対照群では、無作為割り付けから3ヶ月の時点でエンドポイントのアセスメントが行われる。

質問紙はwebアンケートで実施される。1週間以内に回答がない場合には、メールで回答を依頼する。それでも回答がない場合には、再度メールで回答を依頼する。

6ヶ月フォローアップ

介入群・対照群のいずれも、無作為割り付けから6ヶ月後に研究ウェブサイトのURLについての情報を含んだメールを受け取る。質問紙はwebアンケートで実施される。1週間以内に回答がない場合には、メールで回答を依頼する。それでも回答がない場合には、再度メールで回答を依頼する。

# 6. 解析

## 6.1. データ解析

### 6.1.1. 中間モニタリング

中間解析は行われない。

### 6.1.2. 予備的解析

解析の第1歩は、想定される範囲外の値と、論理的に矛盾する部分をチェックすることである。

各アウトカム変数において要約統計量(平均、標準偏差、最小値、最大値、歪度)を算出し、各アウトカムの分布図を得るために、ヒストグラム・プロットを行う。これをベースライン、3ヶ月後、6ヶ月後のそれぞれについて行う。

### 6.1.3. ベースライン・データの解析

BDI-IIやHPQといった連続変数や順序変数については、対照群と介入群の平均値を求め、t検定により群間に有意差があるかどうかを検証する。ベースラインのすべての2値変数(はい/いいえ)については、2群において「はい」である割合を求め、自由度1のχ^2^検定を行う。ベースラインの群間比較を行う“Table 1”では、p値を報告するかもしれないし、報告しないかもしれない。投稿する雑誌によって報告を求めるところとそうでないところがあるため、p値を報告するか否かは投稿する雑誌による。

### 6.1.4. プライマリ・アウトカムの解析

2つのプライマリ・アウトカム、抑うつ重症度の測度であるBDI-IIは3,6ヶ月フォローアップで、大うつ・小うつエピソードは6ヶ月フォローアップでのみ測定される。

BDI-IIは連続変数/順序変数であるため、対照群と介入群の2群で平均値を算出する。大うつエピソードは離散変数であるため対照群と介入群の2群で頻度を算出する。

はじめにt検定またはカイ二乗検定を用いて調整なしの解析を行う。その後、いくつかの調整解析を行う。第1には、ベースラインの差を調整して、3,6ヶ月フォローアップの平均値または頻度を比較するために、ANCOVAまたは多重ロジスティック回帰を行う。また、その他のベースラインの変数を統制する調整解析も同様に行うが、統制群と介入群との間で有意な差がある変数と、解析対象となるアウトカムの強い予測因子となる変数を含む。

これらの解析は、3,6ヶ月フォローアップを完了した対象者に限られる(上記の統計解析方法ではすべて、アウトカムについて欠損値のある対象者は解析対象外となる)。真のintent-to-treat分析では、無作為割り付けされたすべての対象者のデータをもとに治療効果を推定するのであって、フォローアップを完了した対象者のみを対象とするものではないが、すべての対象者についての治療効果を推定するために、階層的線形(混合)モデル(HLM)による混合ランダム係数モデルの分析を行う。対象者は、様々な切片(ベースラインの値)と様々な傾き(ベースラインから3,6ヶ月への変化率)を取るランダム要因となる。データセットは対象者1名あたり3つのレコードから構成される：ひとつはベースライン、のこりは3,6ヶ月フォローアップ。

ベースラインでしか回答しなかった対象者も解析に含められ、各群の3,6ヶ月後の平均値の推定において考慮される。このようなモデルはSPSSやSAS、STATAといった標準的な統計パッケージで適用可能である。

回答者のみの解析に基づく結論と、intent-to-treat 解析に基づく結論が異なっていた場合には、intent-to-treat 解析の結果を報告する。

### 6.1.5. セカンダリ・アウトカムの解析

連続変数/順序変数のアウトカムも、上記と同様の方法で扱われる：t検定により群平均を調整なしで比較し、その後、アウトカムのベースライン値を調整したANCOVAによる群平均の比較を行い、最後にその他のベースライン変数を調整した回帰分析を行う。群平均を階層的線形(混合)モデル(HLM)を用いて比較する。

二値変数(はい/いいえ)については、対照群と介入群の「はい」の割合を比較するが、まずは調整なしの解析を行うためにχ^2^検定を用いる。その後、アウトカム変数のベースラインのみを調整したANCOVAタイプの解析を行うために、ロジスティック回帰分析を行う。最後に、他のベースライン変数を統制した調整ロジスティック回帰分析を行う。

### 6.1.5. 研究からの脱落についての解析

3,6ヶ月フォローアップ完了者と非完了者との間で、ベースラインにおける特性を比較する。3,6ヶ月フォローアップで回答しなかった対象者が、何か重要な傾向を以て回答した対象者と違いがあるのかどうかが知りたいところである。たとえば、非完了者がベースラインにおいてより重症であるかどうかについて知ることは重要である。もしそうであるならば、回答率が対照群と介入群との間で異なるとき特に、研究からの脱落は結果にバイアスをもたらすだろう。

Table 1のすべての変数と、プライマリ・アウトカム、セカンダリ・アウトカムについて、フォローアップ完了者と非完了者の割合を算出する。完了者と非完了者における、連続変数/順序変数の平均値を比較するためにt検定を用いる。二値変数(はい/いいえ)については、自由度1のχ^2^検定において、両者の割合を比較する。

## 6.2. 臨床試験終了の方針

リクルートは2週間を予定している。その後、6ヶ月のフォローアップが開始となる。治療群・待機群ともに無作為割り付けから6ヶ月後のフォローアップとなる。この6ヶ月フォローアップ評価の後、待機群でiCBTを希望する対象者は、iCBTを受けることができる。この追加iCBTについては、付加的な研究となる。

# 7. 組織

## 7.1. プロジェクト・メンバー

### 7.1.1. プロジェクト管理チーム

研究責任者

川上憲人,Norito KAWAKAMI, MD, MPH (精神保健疫学者)

今村幸太郎, Kotaro IMAMURA, MA, MPH (臨床心理士)

島津明人, Akihito SHIMAZU, PhD (臨床心理士)

古川壽亮, Toshi A. FURUKAWA, MD, PhD (精神科医)

松山　裕, Yutaka MATSUYAMA, PhD (生物統計学者)

臨床研究コーディネーター

今村幸太郎, Kotaro IMAMURA, MA, MPH (臨床心理士)

上田芙基子

### 7.1.2. データ管理センター

東京大学大学院医学系研究科精神保健学分野に設置されたデータ管理センターには今村が属する。

### 7.1.3. セラピストとスーパーバイザー

スーパーバイザー

川上憲人

今村幸太郎

iCBTトレーナー

修士、博士、ポスドクレベルの心理士５人を募集する

### 7.1.4. 統計解析部門

川上憲人が統計解析についての責任を担う。

松山は、研究デザインや実施、どのように解析を行い、どのように結果を解釈すべきかについて、また、公刊に向けての結果の報告について、川上と協議する。

## 8.2.研究の実施

### 8.2.1. 運営委員会と下位委員会

プロジェクト管理チームは進捗を確認するため、毎月打ち合わせを行う。

### 8.2.2. データ・安全管理委員会(Data and Safety Monitoring Board :DSMB)

DSMBはCRC、松山( 生物統計学者)、本研究には関与していない者(島津)から構成される。

DSMBは参加者が無作為割り付けされてから3ヶ月ごとに打ち合わせを行う。打ち合わせの目的はCRCが同意により用意された報告を確認することである。

CRCはリクルート状況、データ収集状況(回収率)、有害事象およびその対応方法について確認するための報告を行う。

自殺念慮その他、抑うつの増悪が生じた場合を想定し、参加者が利用できるメールおよび電話番号を用意する。

### 8.2.3. 財務組織

　本研究は、学術振興会科学研究費基盤（Ａ）により実施される。

# 文献

American Psychiatric Association. (2000). *Diagnostic and statistical manual of mental disorders: DSM-IV-TR®*: American Psychiatric Pub.

Andrews, G., Cuijpers, P., Craske, M. G., McEvoy, P., & Titov, N. (2010). Computer therapy for the anxiety and depressive disorders is effective, acceptable and practical health care: a meta-analysis. *PLoS One, 5*(10), e13196. doi: 10.1371/journal.pone.0013196

Beck, A. T. (1967). *Depression: clinical, experimental, and theoretical aspects*. New York,: Hoeber Medical Division.

Beck, A. T. (1979). *Cognitive therapy of depression*. New York: Guilford Press.

Beck, A. T., Steer, R. A., & Brown, G. K. (1996). *BDI-II, Beck depression inventory : manual* (2nd ed.). San Antonio, Tex. Boston: Psychological Corp. ; Harcourt Brace.

Beck, A. T., Ward, C. H., Mendelson, M., Mock, J., & Erbaugh, J. (1961). An inventory for measuring depression. *Arch Gen Psychiatry, 4*, 561-571.

Cuijpers, P., Munoz, R. F., Clarke, G. N., & Lewinsohn, P. M. (2009). Psychoeducational treatment and prevention of depression: the "Coping with Depression" course thirty years later. *Clin Psychol Rev, 29*(5), 449-458. doi: 10.1016/j.cpr.2009.04.005

Cuijpers, P., Smit, F., & van Straten, A. (2007). Psychological treatments of subthreshold depression: a meta-analytic review. *Acta Psychiatr Scand, 115*(6), 434-441. doi: 10.1111/j.1600-0447.2007.00998.x

Cuijpers, P., van Straten, A., Smit, F., Mihalopoulos, C., & Beekman, A. (2008). Preventing the onset of depressive disorders: a meta-analytic review of psychological interventions. *Am J Psychiatry, 165*(10), 1272-1280. doi: 10.1176/appi.ajp.2008.07091422

Furukawa, T. A., Kawakami, N., Saitoh, M., Ono, Y., Nakane, Y., Nakamura, Y., . . . Kikkawa, T. (2008). The performance of the Japanese version of the K6 and K10 in the World Mental Health Survey Japan. *Int J Methods Psychiatr Res, 17*(3), 152-158. doi: 10.1002/mpr.257

Furukawa, T. A., Kessler, R. C., Slade, T., & Andrews, G. (2003). The performance of the K6 and K10 screening scales for psychological distress in the Australian National Survey of Mental Health and Well-Being. *Psychol Med, 33*(2), 357-362.

Gega, L., Marks, I., & Mataix-Cols, D. (2004). Computer-aided CBT self-help for anxiety and depressive disorders: experience of a London clinic and future directions. *J Clin Psychol, 60*(2), 147-157. doi: 10.1002/jclp.10241

Haro, J. M., Arbabzadeh-Bouchez, S., Brugha, T. S., De Girolamo, G., Guyer, M. E., Jin, R., . . . Kessler, R. C. (2006). Concordance of the composite international diagnostic interview version 3.0 (CIDI 3.0) with standardized clinical assessments in the WHO World Mental Health Surveys. *Int J Methods Psychiatr Res, 15*(4), 167-180. doi: Doi 10.1002/Mpr.196

Hiroe, T., Kojima, M., Yamamoto, I., Nojima, S., Kinoshita, Y., Hashimoto, N., . . . Furukawa, T. A. (2005). Gradations of clinical severity and sensitivity to change assessed with the Beck Depression Inventory-II in Japanese patients with depression. *Psychiatry Res, 135*(3), 229-235. doi: 10.1016/j.psychres.2004.03.014

Hosman, C., Jané Llopis, E., & Saxena, S. (2004). *Prevention of mental disorders: Effective interventions and policy options: Summary report*: Geneva: World Health Organization.

Hutchinson, K. H. (1949). An Experiment in the Use of Comics as Instructional Material. *Journal of Educational Sociology, 23*(4), 236-245. doi: Doi 10.2307/2264559

Kawakami, N., Takeshima, T., Ono, Y., Uda, H., Hata, Y., Nakane, Y., . . . Kikkawa, T. (2005). Twelve-month prevalence, severity, and treatment of common mental disorders in communities in Japan: preliminary finding from the World Mental Health Japan Survey 2002-2003. *Psychiatry Clin Neurosci, 59*(4), 441-452. doi: 10.1111/j.1440-1819.2005.01397.x

Kawakami, N., Takeshima, T., Ono, Y., Uda, H., Nakane, Y., Nakamura, Y., . . . Watanabe, M. (2008). Twelve-month prevalence, severity, and treatment of common mental disorders in communities in Japan: the World Mental Health Japan 2002-2004 survey. *The WHO world mental health surveys: global perspectives on the epidemiology of mental disorders*, 474-485.

Kessler, R. C., Akiskal, H. S., Ames, M., Birnbaum, H., Greenberg, P., Hirschfeld, R. M., . . . Wang, P. S. (2006). Prevalence and effects of mood disorders on work performance in a nationally representative sample of U.S. workers. *Am J Psychiatry, 163*(9), 1561-1568. doi: 10.1176/appi.ajp.163.9.1561

Kessler, R. C., Ames, M., Hymel, P. A., Loeppke, R., McKenas, D. K., Richling, D. E., . . . Ustun, T. B. (2004). Using the World Health Organization Health and Work Performance Questionnaire (HPQ) to evaluate the indirect workplace costs of illness. *Journal of Occupational and Environmental Medicine, 46*(6), S23-S37. doi: DOI 10.1097/01.jom.0000126683.75201.c5

Kessler, R. C., Andrews, G., Colpe, L. J., Hiripi, E., Mroczek, D. K., Normand, S. L., . . . Zaslavsky, A. M. (2002). Short screening scales to monitor population prevalences and trends in non-specific psychological distress. *Psychol Med, 32*(6), 959-976.

Kessler, R. C., Barber, C., Beck, A., Berglund, P., Cleary, P. D., McKenas, D., . . . Wang, P. (2003). The world health organization health and work performance questionnaire (HPQ). *Journal of Occupational and Environmental Medicine, 45*(2), 156-174. doi: DOI 10.1097/01.jom.0000052967.43131.51

Kessler, R. C., Barker, P. R., Colpe, L. J., Epstein, J. F., Gfroerer, J. C., Hiripi, E., . . . Zaslavsky, A. M. (2003). Screening for serious mental illness in the general population. *Arch Gen Psychiatry, 60*(2), 184-189.

Kessler, R. C., & Ustun, T. B. (2004). The World Mental Health (WMH) Survey Initiative Version of the World Health Organization (WHO) Composite International Diagnostic Interview (CIDI). *Int J Methods Psychiatr Res, 13*(2), 93-121.

Power, M. J., Katz, R., McGuffin, P., Duggan, C. F., Lam, D., & Beck, A. T. (1994). The Dysfunctional Attitude Scale (DAS): A Comparison of Forms A and B and Proposals for a New Subscaled Version. *Journal of Research in Personality, 28*(3), 263-276. doi: <http://dx.doi.org/10.1006/jrpe.1994.1019>

Richardson, K. M., & Rothstein, H. R. (2008). Effects of occupational stress management intervention programs: a meta-analysis. *J Occup Health Psychol, 13*(1), 69-93. doi: 10.1037/1076-8998.13.1.69

Robins, L. N., Wing, J., Wittchen, H. U., Helzer, J. E., Babor, T. F., Burke, J., . . . Towle, L. H. (1988). The Composite International Diagnostic Interview - an Epidemiologic Instrument Suitable for Use in Conjunction with Different Diagnostic Systems and in Different Cultures. *Arch Gen Psychiatry, 45*(12), 1069-1077.

Ruwaard, J., Lange, A., Bouwman, M., Broeksteeg, J., & Schrieken, B. (2007). E-mailed standardized cognitive behavioural treatment of work-related stress: a randomized controlled trial. *Cogn Behav Ther, 36*(3), 179-192. doi: 10.1080/16506070701381863

Saarni, S. I., Suvisaari, J., Sintonen, H., Pirkola, S., Koskinen, S., Aromaa, A., & Lonnqvist, J. (2007). Impact of psychiatric disorders on health-related quality of life: general population survey. *Br J Psychiatry, 190*, 326-332. doi: 10.1192/bjp.bp.106.025106

Sakurai, K., Nishi, A., Kondo, K., Yanagida, K., & Kawakami, N. (2011). Screening performance of K6/K10 and other screening instruments for mood and anxiety disorders in Japan. *Psychiatry Clin Neurosci, 65*(5), 434-441. doi: 10.1111/j.1440-1819.2011.02236.x

Shimazu, A., Schaufeli, W. B., Kosugi, S., Suzuki, A., Nashiwa, H., Kato, A., . . . Goto, R. (2008). Work engagement in Japan: Validation of the Japanese version of the Utrecht Work Engagement Scale. *Applied Psychology-an International Review-Psychologie Appliquee-Revue Internationale, 57*(3), 510-523. doi: DOI 10.1111/j.1464-0597.2008.00333.x

Sones, W. W. D. (1944). The Comics and Instructional Method. *Journal of Educational Sociology, 18*(4), 232-240. doi: Doi 10.2307/2262696

Spek, V., Cuijpers, P., Nyklicek, I., Riper, H., Keyzer, J., & Pop, V. (2007). Internet-based cognitive behaviour therapy for symptoms of depression and anxiety: a meta-analysis. *Psychol Med, 37*(3), 319-328. doi: 10.1017/S0033291706008944

Tajima, M., Akiyama, T., Numa, H., Kawamura, Y., Okada, Y., Sakai, Y., . . . Power, M. J. (2007). Reliability and validity of the Japanese version of the 24-item Dysfunctional Attitude Scale. *Acta Neuropsychiatrica, 19*(6), 362-367. doi: DOI 10.1111/j.1601-5215.2007.00203.x

Titov, N., Andrews, G., & Sachdev, P. (2010). Computer-delivered cognitive behavioural therapy: effective and getting ready for dissemination. *F1000 Med Rep, 2*. doi: 10.3410/M2-49

van der Klink, J. J., Blonk, R. W., Schene, A. H., & van Dijk, F. J. (2001). The benefits of interventions for work-related stress. *Am J Public Health, 91*(2), 270-276.

World Health Organization. Department of Mental Health and Substance Abuse. (2001). *Atlas: Mental Health Resources in the World 2001*. Geneva: World Health Organization.

World Health Organization. Department of Mental Health and Substance Abuse. (2005). *Mental health atlas 2005* (Rev ed.). Geneva: World Health Organization.
