# Supplementary material for: Effects of an Internet-Based Cognitive Behavioral Therapy (iCBT) Program in Manga Format on Improving Subthreshold Depressive Symptoms among Healthy Workers: A Randomized Controlled Trial
Source: PLoS One. 2014 May 20;9(5):e97167. doi: 10.1371/journal.pone.0097167 (PMC4028193; doi:10.1371/journal.pone.0097167)
Supplement: Protocol S2 — English translation of the original Japanese protocol. (DOCX) [file pone.0097167.s003.docx]

**Effects of an Internet-based cognitive behavioral therapy (iCBT) program among healthy workers: A randomized controlled trial**

The Short title: Internet-based CBT among workers

Norito KAWAKAMI, Kotaro IMAMURA, Toshi A. FURUKAWA, Yutaka MATSUYAMA, Akihito SHIMAZU, Rino UMANODAN, Sonoko KAWAKAMI, Kiyoto KASAI, Fukiko UEDA

This trial will be registered at the UMIN Clinical Trials Registry (UMIN-CTR) (ID = UMINxxxxxxxxx)

# 1. BACKGROUND

Depressive disorder is one of the most prevalent psychiatric disorders, affecting around 340 million people worldwide ([Hosman, Jané Llopis, & Saxena, 2004](#_ENREF_15)), and is associated with a substantial deterioration in quality of life and economic loss in the community and workplace([Kessler et al., 2006](#_ENREF_19); [Saarni et al., 2007](#_ENREF_29)). Thus, the primary prevention of depressive disorder is an important strategy for global mental health.

A growing body of literature has shown that the intervention programs based on cognitive behavioral therapy (CBT) ([Aaron T. Beck, 1967](#_ENREF_3), [1979](#_ENREF_4)) are effective on improving depression and anxiety among workers. A meta-analysis reported that the estimated effect of CBT was Cohen’s d = 0.68 ([van der Klink, Blonk, Schene, & van Dijk, 2001](#_ENREF_36)). Another meta-analysis reported that the estimated effect of CBT was Cohen’s d = 1.16 ([Richardson & Rothstein, 2008](#_ENREF_26)). Moreover, CBT could be an effective approach for preventing depressive disorder. Two previous meta-analyses have shown that CBT is an effective preventive measure for major depressive disorder ([Cuijpers, Munoz, Clarke, & Lewinsohn, 2009](#_ENREF_7); [Cuijpers, van Straten, Smit, Mihalopoulos, & Beekman, 2008](#_ENREF_9)). However, the implementation of these interventions was still limited, because such a program requires professionals well trained in CBT ([World Health Organization. Department of Mental Health and Substance Abuse., 2001](#_ENREF_37), [2005](#_ENREF_38)). In addition, even if a service is provided, the time, cost, and stigma related to mental health treatment could serve as barriers to access to the effective treatment ([Ruwaard, Lange, Bouwman, Broeksteeg, & Schrieken, 2007](#_ENREF_28)).

An innovative way to deliver CBT-based treatment widely is with a computerized CBT (CCBT) and via the Internet CCBT (iCBT). The CCBT and iCBT programs teach basic information and skills based on the same CBT principles as face-to-face CBT programs do, with a highly structured format comprising educational lessons, homework assignments, and supplementary resources ([Titov, Andrews, & Sachdev, 2010](#_ENREF_35)). Previous studies have shown a significant positive treatment effect of CCBT and iCBT programs on depression and anxiety in the clinical setting ([Andrews, Cuijpers, Craske, McEvoy, & Titov, 2010](#_ENREF_2)). An iCBT program is particularly beneficial with its high anonymity ([Gega, Marks, & Mataix-Cols, 2004](#_ENREF_12)) and high accessibility ([Spek et al., 2007](#_ENREF_33)).

However, no iCBT program is available that would target specifically the general working population, the effectiveness of which would be tested with an randomized controlled trial (RCT). In addition, no previous RCT investigated a beneficial effect of an iCBT program on reducing a risk of depressive disorder which was diagnosed according to standardized diagnostic criteria, such as the DSM-IV ([American Psychiatric Association., 2000](#_ENREF_1)).

The purpose of this randomized controlled study is as follows:

1. to develop a new six-week iCBT program for workers who has subthreshold depressive symptoms in Japan.
2. to examine the effects of the iCBT program on improving the symptoms of depression at three- and six-month follow-ups among workers who has subthreshold depressive symptoms in Japan.
3. to decrease the risk of DSM-IV major depressive episode (MDE) through the six-month follow-up among workers in Japan.
4. to examine the effects of the iCBT program on improving work engagement and work performance at three- and six-month follow-ups among workers in Japan.

The study will be a two-arm, parallel-group, TAU-controlled, non-blinded randomized study.

# 2. OBJECTIVES

## 2.1 Primary question

Our primary question is:

“Among workers with subthreshold depression, will an Internet-based CBT program consisting of cognitive restructuring, assertiveness training, and problem-solving techniques in addition to the standard Employee Assistance Program (EAP) outperform EAP alone, in terms of depression severity (measured with BDI-II) and the onset of major depressive episode (measured with the web-version of the Japanese WHO-CIDI 3.0) at the end of the follow-up survey?”

The primary outcome measures in this study will therefore be:

1. depression severity at three- and six -month post-randomization as measured by the BDI-II
2. the onset of MDE during the 6-month follow-up as measured by the web-version of the Japanese WHO-CIDI 3.0

These two primary outcomes will be used to decide whether or not the intervention was effective.

## 2.2. Secondary questions

Secondary outcomes will include:

At three- and six-month post-randomization:

1. depression severity as measured by the Kessler’s psychological distress scale (K6).
2. work performance.
   1. “Effective hours worked” for the past month as measured by the one item from the WHO Health and Work Performance Questionnaire (HPQ).
   2. the number of Sick leave days during past 3 month.
3. work engagement as measured by the 9 Utrecht Work Engagement Scale (UWES).
4. depressogenic schemata as measured by the 24-item Dysfunctional Attitude Scale (DAS-24).
5. improvement of knowledge and self-efficacy regarding five components of the iCBT program (Stress management, Cognitive restructuring, Assertive communication, Problem-solving and Relaxation training) are scored on 5-point scale ranging from 0 (none) to 4 (enough).
   1. knowledge improvement as assessed by asking participants, “How much knowledge do you have about…”
   2. self-efficacy improvement as assessed by asking respondents “How confident are you that you can do….”
6. participant satisfaction as measured by the visual analog scale for satisfaction and a detailed ad-hoc questionnaire for those who received iCBT at the end of the intervention program.

## 2.3. Subgroup hypotheses

The effectiveness of the program may differ according to the initial severity of depression. We will therefore use as one stratification factor high/low subthreshold depression (i.e., participants who scored 5 or more for the K6) at baseline survey, and analyze the results according to these a priori defined subgroups (selective intervention effect), while a sub-analysis will be done for all subjects including those with K6 scores of 0-4, as well. We will also analyze the results according to whole sample (universal intervention effect).

## 2.4. Adverse effects

We expect no adverse health effect from this intervention, except possibly for deterioration in depression (and, ultimately, suicide). We will provide the emergency phone call number and e-mail address at the central office, should any serious suicidal wish occurs. The clinical research coordinator (CRC; Kotaro IMAMURA), who is a clinical psychologist (MS, MPH), will then deal with the emergency call or e-mail first by himself, and then consult with the clinical supervisors (Norito KAWAKAMI, MD, PhD) to provide appropriate care.

We will measure deterioration in depression in our primary and secondary outcomes. We will measure suicidal attempt, completed suicide and hospitalization at six-month follow-up. We will not implement “stopping decision rules” for this study, because we will not have a sample large enough to determine whether adverse events are clearly related to study participation or if outcome data are sufficient to answer our primary study questions prior to enrollment of the full sample.

Professor KAWAKAMI, the Principal Investigator, will be responsible for all the decisions. Professor KAWAKAMI will be available for review of all instances of reported adverse effects and suicide risk within 24 hours. When he is not available to fulfill this role because of travel or other obligations, another designated clinical investigator will be appointed to monitor participant safety.

# 3. PARTICIPANTS

## 3.1. Study population

Working men and women with subthreshold depression will be selected according to the following criteria:

### 3.1.1. Inclusion criteria

1. Age 20-60 at study entry
2. Men and women
3. Currently employed full-time by the business company
4. K6 scores greater than or equal to 5 at screening (while a sub-analysis will be done for all subjects including those with K6 scores of 0-4, as well)
5. Can access the Internet via a PC at home or at workplace

### 3.1.2. Exclusion criteria

1. Non-regular or part-time employees.
2. Sick leave for 15 or more days for a physical or mental condition in the past 3 months.
3. Current treatment for a mental health problem from a mental health professional.
4. Having major depressive episode in the past a month, as ascertained by the web-version of the Japanese WHO-CIDI 3.0.
5. Having lifetime history of bipolar disorder, as ascertained by the web-version of the Japanese WHO-CIDI 3.0.

All workers in company A (N=290) and all workers who belonged to three selected departments (N=about 1,500) at the headquarter of company B (the total employee size, c.a. 11,000) will be recruited by an invitation e-mail.

## 3.2. Sample size assumptions and estimates

A systematic review of psychological treatments, mostly CBT, for subthreshold depression yielded a Cohen’s d of 0.42 (95%CI: 0.23 to 0.60) at post-test ([Cuijpers, Smit, & van Straten, 2007](#_ENREF_8)). (a Cohen's d of .42 indicates a treatment effect (difference between control mean and intervention mean on the outcome measure) equal to 0.42 standard deviations (where standard deviation(SD) of interest if the SD of the outcome measure)

| Alpha | power | Effect size | N in each cell  (The allocation ratio = 1:1) | Total N |
| --- | --- | --- | --- | --- |
| 0.05 | 0.90 | 0.4 | 131 | 262 |
| 0.05 | 0.80 | 0.4 | 98 | 196 |

Thus, if we randomize 131 in the intervention group and control group for a total of 262 randomized, we will have 90% power to detect a treatment effect assuming that this difference is equal to .40 standard deviations. However, these calculations ignore dropout. We expect that 75% will complete our six-month follow-up, resulting in 98 respondents in the intervention group and control group at six-month. In this situation, we will have 90% power to detect an effect size equal to .47 SD, which is exactly equal to our best estimate of what effect we expect to see at month 4 in this trial.

## 3.3. Recruitment of participants

The company representatives and/or the principal investigator will visit study sites and meet with the employees, if possible in small groups, to explain the purpose and rough procedures of the study to increase interest for this program beforehand.

CRC will send out invitation mail to 1700 employees, of whom 680 will give the informed consent, of whom 272 will be candidates (K6>=5), of whom 245 will be eligible. These 245 will be randomized to intervention group (n=122) or to control group (n=123). They will be allowed to complete the six lessons within 10 weeks after the baseline survey. Those on the control group can receive the iCBT only after six-months.

### 3.3.1. Assessment of eligibility

We will sent an invitation e-mail to all workers who belongs to company A or three selected departments at the headquarter of company B. Assessment of eligibility will be checked at the baseline survey. We prepared a website which contained full explanation of the study. Before the baseline survey, participants were invited to read the explanation on the website, and asked to click an “agree” button to show their consent to participate in the study; then they proceeded to the baseline questionnaire page.

Those who were found to be currently in major depressive episode will be excluded from this trial but will be contacted by the CRC, so that they would be advised to see an industrial physician or an extra-mural specialist. The CRC would explain by e-mail that, according to all the data accumulated so far, the subject was judged to profit more by seeing a specialist in person than by this iCBT.

### 3.3.2. Baseline assessments

Eligibility assessments will be combined with baseline survey. The baseline survey will therefore consist of the following variables.

1. sex
2. age
3. occupational, educational and marital statuses.
4. K6 score past a month
5. BDI-II score past two weeks
6. Work engagement
7. Health and Productivity Questionnaire
   - Total hours worked for the past 4 weeks, Relative absenteeism for the past 4 weeks (% of the working hours absent), Relative presenteeism for the past 4 weeks
8. Current treatment for physical and/or mental conditions
9. Any sick leave in the three months
10. CIDI results for mood disorders

### 3.3.3. Informed consent

We prepared a website which contained full explanation of the study. Before the baseline survey, participants were invited to read the explanation on the website, and asked to click an “agree” button to show their consent to participate in the study; then they proceeded to the baseline questionnaire page.

Candidates will be informed that their participation is totally voluntary, that even after voluntarily participating they can withdraw from the intervention or from both the intervention and the assessment at any time without stating the reason, and that neither participation nor withdrawal will lead to any advantage or disadvantage at the company. Data of each participant will be handled with sequentially allocated numbers to keep the participant’s confidentiality and kept in a computer not connected to the Internet. It will be accessible to the CRC only.

### 3.3.4. Intervention allocation

We will randomize eligible subjects to intervention and control groups at a ratio of 1:1. Randomization will be stratified on two variables, namely depression severity at baseline (K6=<4 vs. >=5) and center (company A or company B). Assuming that subjects are recruited from 2 sites, there will be a total of 4 strata. The study statistician will prepare 4 lists of random assignments ahead of time, one for each of the 4 strata. Each list will be blocked to ensure that the ratio of intervention to control is exactly 1:1 at set intervals within each stratum. The statistician will send these lists to the research assistant who is not involved in any other aspects of the study and who will build a spreadsheet that enables concealed sequential allocation and will be responsible for keeping these lists in the safe place.

Before randomizing an individual, the CRC will make a final determination of whether or not the person is eligible to be in the randomized trial. For example, prior to randomization, we will exclude those who having major depressive episode in the past a month. Once the CRC determines the eligibility of the participant, she will enter his/her registration number and the stratification variables to the spreadsheet which will automatically generate the allocation according to the pre-determined random sequence. The entered variables cannot be undone in order to guarantee allocation concealment.

Those randomized to control group will be put on a waiting list and after six-month can receive the iCBT if they wish to do so.

One stratification factor is K6>=5 and K6<=4. The cutoff score for K6 selected based on a previous report, which suggested that 4/5 is the optimal cutoff point for K6 in terms of discriminating community residents and patients with mood and anxiety disorders ([Sakurai, Nishi, Kondo, Yanagida, & Kawakami, 2011](#_ENREF_30)).

# 4. INTERVENTIONS

## 4.1. Description and schedule

In order for the randomization and start of intervention to be as close as possible, CRC will endeavor to randomize the participant immediately after closing for application and send the e-mail about iCBT program course description as soon as possible.

Participants who will be allocated to the intervention group studied the new iCBT program called the Internet CBT program; Useful mental health solutions series for business. The program is a six-week web-based training course to provide stress management skills. This program is structured into six lessons, with one lesson given per week. Learning one lesson required about 30 minutes, including homework. This program can be used anywhere the Internet is available.

One of the unique features of the program is that the training is provided along with a Manga (Japanese comic) story of a psychologist and a client worker to facilitate the understanding of the participants. Several merits of using a comic story and comic characters in education have been acknowledged through research. First, these materials help motivate individuals, and high motivation is useful to keep participants in the program ([Hutchinson, 1949](#_ENREF_16)). Second, they facilitate easy learning. A program with text combined with comic stories would be easier for a learner to understand compared to a text-only program ([Hutchinson, 1949](#_ENREF_16); [Sones, 1944](#_ENREF_32)). Third, comic stories foster learners’ interest in the program ([Sones, 1944](#_ENREF_32)). These merits might be applicable to education in the workplace, because most Japanese people of working age are familiar with comics.

In terms of the CBT components of the program, included are self-monitoring skills, cognitive restructuring skills, assertiveness, problem-solving skills, and relaxation skills. At the end of each lesson, participants are asked to submit homework to facilitate their understanding, although it is voluntary. Participants who will submit their homework will receive feedback from trained staff (clinical psychologists).

Participants will be allowed to complete the six lessons within 10 weeks after the baseline survey. Participants will be reminded by e-mail to complete each lesson if they had not already done so. Reminders will be sent from the research office to the participants every Monday.

The EAP program is the one which has already been in use by this company. The program includes a web site for mental and physical health, a p.r.n. telephone or in-person counseling service accessible to all the employees and on-site lectures on mental and physical health for managers and workers.

Participants in the control group will also receive an e-mail message once a month titled “Useful information for stress management.” Each e-mail message consists of about 500 words in Japanese and included stress management tips. The contents are as follows: 1) How to have a good sleep, 2) The effects of your diet and ingesting alcohol for stress, 3) How to have a good holiday, 4) Relaxing by listening to music and taking a bath, and 5) The effects of exercise on stress.

## 4.2. Quality control of treatments

The iCBT program to be used in this study was developed by the investigators based on cognitive-behavioral theory. The exactly same content will be provided to all participants in the intervention group at each session through a web-based learning management system set up at a server of the Department of Mental Health, University of Tokyo.

Concerning tailored feedback comments for a homework which a respondent submits, the homework will be reviewed and commented on by five psychologists at MSc, PhD or post-PhD level. To ensure a similar quality of the feedback comments, a brief guidance and half-day training will be given to these psychologists face to fade by the CRC (Kotaro IMAMURA), who is also nan experienced clinical psychologist, with a written manual with several examples. All feedback comments to homeworks will be recorded and reviewed by the CRC to monitor the psychologists’ performance and provide additional training to them if necessary.

## 4.3. Measures of client adherence

The CRC will check for client adherence and send a reminded e-mail to the participant if any of the following is noticed:

1. More than a week have elapsed since the announcement by e-mail about iCBT program
2. More than a week have elapsed between sessions

# 5. ASSESSMENTS

## 5.1. Measures

### Beck Depression Inventory-II (BDI-II)

The Beck Depression Inventory, originally published in 1961 ([A. T. Beck, Ward, Mendelson, Mock, & Erbaugh, 1961](#_ENREF_6)), has been the most widely used self-report measure of depression severity. With the advent of the DSM-IV, the time frame and question items have been updated as the 2nd edition of the BDI, and its reliability and validity have been confirmed ([Aaron T. Beck, Steer, & Brown, 1996](#_ENREF_5)). The reliability and validity of the Japanese version have been found to be excellent ([Hiroe et al., 2005](#_ENREF_14)).

We will administer BDI-II at baseline, at three-month follow-up (end of acute phase treatment) and at six-month follow-up.

### Composite International Diagnostic Interview (CIDI)

The CIDI is a widely used fully-structured diagnostic interview for assessing mental disorders, to be used with the general population by trained lay interviewers ([Robins et al., 1988](#_ENREF_27)). It has been successively updated to accommodate the DSM-IV. We used the most recent computerized version ([Kessler & Ustun, 2004](#_ENREF_24)) and administered the sections for mood disorders. The concordance between the CIDI and standardized clinical assessments has been reported ([Haro et al., 2006](#_ENREF_13)). The Japanese version of the HPQ and CIDI has been used in the World Mental Health Survey in Japan ([Kawakami et al., 2005](#_ENREF_17)).

The face-to-face version of WHO-CIDI 3.0 was translated into Japanese and tested its validity for diagnosing MDE ([Kawakami et al., 2008](#_ENREF_18)).

### Kessler’s psychological distress scale (K6)

K6 is a recently developed very short (6-item) self-report questionnaire to screen for common mental disorders ([Kessler et al., 2002](#_ENREF_21)). It is based on modern item response theory methods and consists of questions that are maximally discriminative of respondents in the 90th-99th percentile range of the general population distribution because it is known that between 5-10% of the population suffer from mental disorders at any point in time. Only items displaying constant psychometric characteristics across sociodemographic variation are included in the final model. K6 has been found to work as well as and better than some widely used screening questionnaires ([Furukawa, Kessler, Slade, & Andrews, 2003](#_ENREF_11); [Kessler, Barker, et al., 2003](#_ENREF_23)). The Japanese version has been validated ([Furukawa et al., 2008](#_ENREF_10)).

We will use K6 as the initial screening instrument. Together with the screening, three-month follow-up and six-month follow-up data (which will all be administered to the control group as well), K6 will be one of the secondary outcome measures.

### Health and Productivity Questionnaire (HPQ)

The World Health Organization health and Productivity Questionnaire (HPQ) is a self-report instrument designed to estimate the workplace costs of health problems in terms of self-reported sickness absence (absenteeism) and reduced job performance (presenteeism). Validation studies have found documented significant associations (r=0.61 to 0.87) of HPQ work hours assessments with payroll records ([Kessler, Barber, et al., 2003](#_ENREF_22)) and job performance assessments with supervisor ratings (r=0.52) ([Kessler et al., 2004](#_ENREF_20)) and other administrative records (area under the curve, 0.58 to 0.72) ([Kessler & Ustun, 2004](#_ENREF_24)).

We will administer HPQ at baseline, at three-month follow-up (end of acute phase treatment) and at six-month follow-up.

### Sick leave days during past 3 month

Respondents will be asked to report the number of Sick leave days during past 3 month.

We will administer the number of Sick leave days during past 3 month at baseline, at three-month follow-up (end of acute phase treatment) and at six-month follow-up.

### Utrecht Work Engagement Scale (UWES)

Work engagement will be assessed using the short form of the Japanese version of the Utrecht Work Engagement Scale (UWES) ([Shimazu et al., 2008](#_ENREF_31)). The UWES consists of 3 subscales comprising 9 items (i.e., vigor, dedication, absorption). Items are scored on a 7-pointscale ranging from 0 (never) to 6 (always). Item examples are “At my job, I feel strong and vigorous” (vigor), “I am enthusiastic about my job” (dedication), and “I am immersed in my work” (absorption). A total score is calculated from all 9 items.

We will administer UWES at baseline, at three-month follow-up (end of acute phase treatment) and at six-month follow-up.

### The 24-item Dysfunctional Attitude Scale (DAS-24)

The 24-item Dysfunctional Attitude Scale (DAS-24) is a short version of the Dysfunctional Attitude Scale, which is a self-report inventory measuring depressogenic schemata. Each item is scored on a scale ranging from one (totally disagree) to seven (totally agree), with a higher score indicating a more dysfunctional attitude ([Power et al., 1994](#_ENREF_25)). The Japanese version has been developed and tested, and its reliability and validity have been established ([Tajima et al., 2007](#_ENREF_34)).

We will administer DAS at baseline, at three-month follow-up (end of acute phase treatment) and at six-month follow-up.

### Improvement of knowledge and self-efficacy

Respondents will be asked to rate their improvement of knowledge and self-efficacy regarding five components of the iCBT program (Stress management, Cognitive restructuring, Assertive communication, Problem-solving, Relaxation training). Knowledge improvement will be assessed by asking participants, “How much knowledge do you have about…” and self-efficacy improvement was assessed by asking respondents “How confident are you that you can do….” Both items were scored on 5-point scale ranging from 0 (none) to 4 (enough).

We will administer the rate of improvement of knowledge and self-efficacy at baseline, at three-month follow-up (end of acute phase treatment) and at six-month follow-up.

## 5.2. Follow-up visit description and schedule

### 5.2.1. Drop out from the intervention

The subject is free to withdraw from the intervention and/or the study at any time. Even if someone discontinues participation in the intervention, we will therefore make every effort to collect outcome data from them at the follow-ups at month three and at month six.

If a new mental condition arises or the existing mental condition worsens, all the participants, whether during the iCBT or outside of the iCBT, are free to utilize the EAP routinely made available by the company and/or seek professional help outside the company. In addition, they can send the e-mail or call up the free hotline connected to the study center psychologist.

If a new mental condition or worsening of the existing subthreshold depression occurs during the iCBT such that treatment outside the current program is advisable, the counselor must advise the subject to seek professional in-person help from the industrial physician, his/her home doctor or a mental health specialist.

The participation in the intervention itself can be continued if the participant see no conflict between the iCBT and the newly offered treatment, or discontinued should there be any perceived conflict or wish to withdraw expressed from the part of the participant. Even if the iCBT itself is discontinued, the participant is encouraged to take part in the three- and six-months follow-ups.

### 5.2.2. Drop out from the study

Those who withdraw consent to the follow-ups at three and/or six months will be considered dropouts from the study.

### 5.2.3. How to enhance adherence and reduce dropouts

Dropout from the intervention will be strictly distinguished and differentiated from dropout from the study. We will ask the client, so far as it is possible with him/her, that he/she respond to the final assessment even if he/she has chosen to stop the iCBT itself.

Non-respondent will receive the reminded e-mail at least two times from the research center during the learning of iCBT and at three- and six-month follow-up surveys.

## 5.3. Assessment of response variables

### 5.3.1. Independence of the self-reports

In this study, all outcomes will be collected on the web-based surveys. CRC can only access the collected data, in order to assure anonymity and independence of the ratings.

### 5.3.2. Data collection

Three-month follow-up (Acute-phase outcomes)

In the intervention group, the primary outcome will be measured at three-month post randomization, i.e. approximately two weeks after the end of the term of learning the iCBT program. In the control group, the endpoint assessment will take place at three-month post randomization.

The outcome data will be collected on the web-based surveys. If no response is received within a week, participants will receive a reminded e-mail asking for completing the web survey. If no response is still available, CRC will send the reminded e-mail to all the participants again.

Six-month follow-up

Both the intervention group and the control group will receive the e-mail which contains information about the URL of research website at six-month post randomization. If no response is received within a week, participants will receive a reminded e-mail asking for completing the web survey. If no response is still available, CRC will send the reminded e-mail to all the participants again.

# 6. ANALYSES

## 6.1. Data analysis

### 6.1.1. Interim monitoring

No interim analysis will be performed.

### 6.1.2. Preliminary Analyses

Our first set of analyses will check for out-of-range values and logical inconsistencies.

We will also compute summary statistics (mean, standard deviation, minimum, maximum, and skewedness) for each of our outcome variables and do a histogram plot to get a graphical picture of the distribution for each outcome. We will do this separately for outcomes at baseline, three- and six-month.

### 6.1.3. Analyses of Baseline Data

For continuous and ordinal variables, such as BD1-II and HPQ, we will compute the means for the control and intervention groups and test whether they are significantly different using a t-test. For all yes/no baseline variables, we will compute the percentage yes in each of the two groups and compare using a 1 degree of freedom chi-square test. We may or may not report the p-values from these tests in our "Table 1", which will compare the groups at baseline. Whether or not we report them will depend on the journal that we submit the main outcome paper to since some journals want them reported and some do not.

### 6.1.4. Analyses of Primary Outcomes

Our two primary outcomes are the BDI-II measure of depression severity and the web-version of the Japanese WHO-CIDI 3.0 measure of the onset of MDE. The BDI-II will be assessed at three- and six-month follow-ups. The Japanese WHO-CIDI 3.0 will be assessed at the six-month follow-up.

The BDI-II is continuous/ordinal variables so will compute the mean score at three- and six-month follow-ups in the two groups, control and intervention. The onset of MDE is discrete variable so will compute the number of incidence at three- and six-month follow-ups in the two groups, control and intervention.

We will first do unadjusted analyses to compare the means using a t-test. We will then do several adjusted analyses. First, ANCOVA or multiple logistic regression analyses will be used to compare the three- and six-month means or the number of incidence on the outcome adjusting for baseline differences on the outcome. We will also do adjusted analyses which control for additional baseline variables, including variables which differ significantly between the control and intervention groups and variables which are strong predictors of the outcome under analysis.

These analyses will be restricted to subjects who complete the three- and six-month follow-ups (all of the statistical methods described above drop from the analysis those with a missing value on the outcome). Since a true intent-to-treat analysis estimates treatment effects based on data from all randomized subjects, not just those who complete a given follow-up, we will also do some mixed-model hierarchical linear models (HLM), using a mixed-model random coefficient model to estimate treatment effects based on all subjects. Subjects are "random effects" with varying intercepts (baseline values) and vary slopes (rates of change from baseline to three- and six-month). The dataset is structured so that each person has up to three records: baseline, three- and six-month follow-ups.

Even subjects who complete only the baseline enter into the analysis and are taken into account when estimating the three- and six-month mean in each group. Such a model can be fit in standard statistical packages such as SPSS, SAS, and STATA.

If conclusions based on the respondent-only and the intent-to-treat analyses are different, we will report intent-to-treat results.

### 6.1.5. Analyses of secondary outcomes

Analyses of continuous/ordinal secondary outcomes will be done in the same way as described above: first unadjusted comparison of groups means using t-tests, then ANCOVA comparisons of group means adjusting for baseline values of the outcome, and finally regression analyses which adjust for additional baseline variables. We may also compare group means using mixed-model HLM analyses.

For yes/no outcome variables, we will compare the percentages yes in the control and intervention group first using a chi-square test to do an unadjusted analysis. We will then use logistic regression to do an ANCOVA type analysis which adjusts only the baseline of the outcome. Lastly we will do adjusted logistic analyses which control for other baseline variables.

### 6.1.5. Analysis of non-response

We will do analyses to compare the baseline characteristics of subjects who complete the three- and six-month follow-ups and subjects who do not complete the three- and six-month follow-ups (completers and non-completers). We want to know if those who do not complete the three- and six-month follow-ups differ in important ways from those who do. For example, it would be important to know if the non-completers have more severe depression at baseline. If so, non-completion would be more likely to bias the results, particularly if the completion rates differ between control and intervention.

For all of our variables in Table 1, we will compute means and percentages of the completers and non-completers for each treatment group at baseline. We will use t-tests to compare the means for continuous/ordinal variables in the completers and non-completers. For yes/no variables, we will compare the percentages in these two groups using a 1 d.f. chi-square test.

## 6.2. Termination policy

We are planning a recruitment phase of two weeks. We will then start the six-month follow-up. After this six-month follow-up evaluation, those in the waiting list group who wish to receive the iCBT program can receive that.

# 7. ORGANIZATION

## 7.1. Participating investigators

### 7.1.1. Project management team

Co-PI

Norito Kawakami, MD, PhD, MPH (psychiatric epidemiologist)

Kotaro IMAMURA, MA, MPH (clinical psychologist)

Akihito Shimazu, PhD (clinical psychologist)

Toshi A. Furukawa, MD, PhD (psychiatrist)

Yutaka MATSUYAMA, PhD (biostatistician)

Clinical research coordinator

Kotaro IMAMURA, MA, MPH (clinical psychologist)

Fukiko UEDA

### 7.1.2. Data coordinating center

Kotaro IMAMUJRA will be at the Data coordinating center to be located within the Department of Mental Health, Graduate School of Medicine, The University of Tokyo.

### 7.1.3. Therapists and supervisors

Supervisors

Dr. Norito KAWAKAMI

Kotaro IMAMURA

iCBT trainers

5 psychologists at MSc, PhD or post-PhD level will be recruited.

### 6.1.4. Statistical unit

Dr. Norito KAWAKAMI will be responsible for the statistical analyses.

Dr. Yutaka MATSUYAMA will consult with Dr. KAWAKAMI regarding the design and conduct of the study, what statistical analyses should be done and how to interpret the results, and the reporting of the results for publication.

## 8.2. Study administration

### 8.2.1. Steering committee

The CRC will summarize the progress of recruitment and data collection every month, and send out the abbreviated report to all the study collaborators. The project management team will meet every month to monitor the progress of the study.

### 8.2.2. Data and Safety Monitoring Board (DSMB)

The DSMB will consist of the CRC, Dr. MATSUYAMA (biostatistician), and Dr. SHIMAZU (who is not involved in this study).

The DSMB will meet every three-month after the first client is randomized. The purpose of the meeting will be to review the report prepared by the CRC.

CRC will prepare for the DSMB reports which monitor recruitment progress and data collection (e.g. % completing different follow-ups) and adverse events and their resolution.

We will prepare a free e-mail address and phone number for participants to call up, should they have any suicidal ideation and other significant worsening of their depression. (Cf. 2.4. Adverse events)

### 8.2.3. Funding organization

Grant-in-Aid for Scientific Research (A) 2009 and 2010 (No. 20240062) obtained from the Ministry of Education, Culture, Sports, Science and Technology, Japan.

# REFERENCES

American Psychiatric Association. (2000). *Diagnostic and statistical manual of mental disorders: DSM-IV-TR®*: American Psychiatric Pub.

Andrews, G., Cuijpers, P., Craske, M. G., McEvoy, P., & Titov, N. (2010). Computer therapy for the anxiety and depressive disorders is effective, acceptable and practical health care: a meta-analysis. *PLoS One, 5*(10), e13196. doi: 10.1371/journal.pone.0013196

Beck, A. T. (1967). *Depression: clinical, experimental, and theoretical aspects*. New York,: Hoeber Medical Division.

Beck, A. T. (1979). *Cognitive therapy of depression*. New York: Guilford Press.

Beck, A. T., Steer, R. A., & Brown, G. K. (1996). *BDI-II, Beck depression inventory : manual* (2nd ed.). San Antonio, Tex. Boston: Psychological Corp. ; Harcourt Brace.

Beck, A. T., Ward, C. H., Mendelson, M., Mock, J., & Erbaugh, J. (1961). An inventory for measuring depression. *Arch Gen Psychiatry, 4*, 561-571.

Cuijpers, P., Munoz, R. F., Clarke, G. N., & Lewinsohn, P. M. (2009). Psychoeducational treatment and prevention of depression: the "Coping with Depression" course thirty years later. *Clin Psychol Rev, 29*(5), 449-458. doi: 10.1016/j.cpr.2009.04.005

Cuijpers, P., Smit, F., & van Straten, A. (2007). Psychological treatments of subthreshold depression: a meta-analytic review. *Acta Psychiatr Scand, 115*(6), 434-441. doi: 10.1111/j.1600-0447.2007.00998.x

Cuijpers, P., van Straten, A., Smit, F., Mihalopoulos, C., & Beekman, A. (2008). Preventing the onset of depressive disorders: a meta-analytic review of psychological interventions. *Am J Psychiatry, 165*(10), 1272-1280. doi: 10.1176/appi.ajp.2008.07091422

Furukawa, T. A., Kawakami, N., Saitoh, M., Ono, Y., Nakane, Y., Nakamura, Y., . . . Kikkawa, T. (2008). The performance of the Japanese version of the K6 and K10 in the World Mental Health Survey Japan. *Int J Methods Psychiatr Res, 17*(3), 152-158. doi: 10.1002/mpr.257

Furukawa, T. A., Kessler, R. C., Slade, T., & Andrews, G. (2003). The performance of the K6 and K10 screening scales for psychological distress in the Australian National Survey of Mental Health and Well-Being. *Psychol Med, 33*(2), 357-362.

Gega, L., Marks, I., & Mataix-Cols, D. (2004). Computer-aided CBT self-help for anxiety and depressive disorders: experience of a London clinic and future directions. *J Clin Psychol, 60*(2), 147-157. doi: 10.1002/jclp.10241

Haro, J. M., Arbabzadeh-Bouchez, S., Brugha, T. S., De Girolamo, G., Guyer, M. E., Jin, R., . . . Kessler, R. C. (2006). Concordance of the composite international diagnostic interview version 3.0 (CIDI 3.0) with standardized clinical assessments in the WHO World Mental Health Surveys. *Int J Methods Psychiatr Res, 15*(4), 167-180. doi: Doi 10.1002/Mpr.196

Hiroe, T., Kojima, M., Yamamoto, I., Nojima, S., Kinoshita, Y., Hashimoto, N., . . . Furukawa, T. A. (2005). Gradations of clinical severity and sensitivity to change assessed with the Beck Depression Inventory-II in Japanese patients with depression. *Psychiatry Res, 135*(3), 229-235. doi: 10.1016/j.psychres.2004.03.014

Hosman, C., Jané Llopis, E., & Saxena, S. (2004). *Prevention of mental disorders: Effective interventions and policy options: Summary report*: Geneva: World Health Organization.

Hutchinson, K. H. (1949). An Experiment in the Use of Comics as Instructional Material. *Journal of Educational Sociology, 23*(4), 236-245. doi: Doi 10.2307/2264559

Kawakami, N., Takeshima, T., Ono, Y., Uda, H., Hata, Y., Nakane, Y., . . . Kikkawa, T. (2005). Twelve-month prevalence, severity, and treatment of common mental disorders in communities in Japan: preliminary finding from the World Mental Health Japan Survey 2002-2003. *Psychiatry Clin Neurosci, 59*(4), 441-452. doi: 10.1111/j.1440-1819.2005.01397.x

Kawakami, N., Takeshima, T., Ono, Y., Uda, H., Nakane, Y., Nakamura, Y., . . . Watanabe, M. (2008). Twelve-month prevalence, severity, and treatment of common mental disorders in communities in Japan: the World Mental Health Japan 2002-2004 survey. *The WHO world mental health surveys: global perspectives on the epidemiology of mental disorders*, 474-485.

Kessler, R. C., Akiskal, H. S., Ames, M., Birnbaum, H., Greenberg, P., Hirschfeld, R. M., . . . Wang, P. S. (2006). Prevalence and effects of mood disorders on work performance in a nationally representative sample of U.S. workers. *Am J Psychiatry, 163*(9), 1561-1568. doi: 10.1176/appi.ajp.163.9.1561

Kessler, R. C., Ames, M., Hymel, P. A., Loeppke, R., McKenas, D. K., Richling, D. E., . . . Ustun, T. B. (2004). Using the World Health Organization Health and Work Performance Questionnaire (HPQ) to evaluate the indirect workplace costs of illness. *Journal of Occupational and Environmental Medicine, 46*(6), S23-S37. doi: DOI 10.1097/01.jom.0000126683.75201.c5

Kessler, R. C., Andrews, G., Colpe, L. J., Hiripi, E., Mroczek, D. K., Normand, S. L., . . . Zaslavsky, A. M. (2002). Short screening scales to monitor population prevalences and trends in non-specific psychological distress. *Psychol Med, 32*(6), 959-976.

Kessler, R. C., Barber, C., Beck, A., Berglund, P., Cleary, P. D., McKenas, D., . . . Wang, P. (2003). The world health organization health and work performance questionnaire (HPQ). *Journal of Occupational and Environmental Medicine, 45*(2), 156-174. doi: DOI 10.1097/01.jom.0000052967.43131.51

Kessler, R. C., Barker, P. R., Colpe, L. J., Epstein, J. F., Gfroerer, J. C., Hiripi, E., . . . Zaslavsky, A. M. (2003). Screening for serious mental illness in the general population. *Arch Gen Psychiatry, 60*(2), 184-189.

Kessler, R. C., & Ustun, T. B. (2004). The World Mental Health (WMH) Survey Initiative Version of the World Health Organization (WHO) Composite International Diagnostic Interview (CIDI). *Int J Methods Psychiatr Res, 13*(2), 93-121.

Power, M. J., Katz, R., McGuffin, P., Duggan, C. F., Lam, D., & Beck, A. T. (1994). The Dysfunctional Attitude Scale (DAS): A Comparison of Forms A and B and Proposals for a New Subscaled Version. *Journal of Research in Personality, 28*(3), 263-276. doi: <http://dx.doi.org/10.1006/jrpe.1994.1019>

Richardson, K. M., & Rothstein, H. R. (2008). Effects of occupational stress management intervention programs: a meta-analysis. *J Occup Health Psychol, 13*(1), 69-93. doi: 10.1037/1076-8998.13.1.69

Robins, L. N., Wing, J., Wittchen, H. U., Helzer, J. E., Babor, T. F., Burke, J., . . . Towle, L. H. (1988). The Composite International Diagnostic Interview - an Epidemiologic Instrument Suitable for Use in Conjunction with Different Diagnostic Systems and in Different Cultures. *Arch Gen Psychiatry, 45*(12), 1069-1077.

Ruwaard, J., Lange, A., Bouwman, M., Broeksteeg, J., & Schrieken, B. (2007). E-mailed standardized cognitive behavioural treatment of work-related stress: a randomized controlled trial. *Cogn Behav Ther, 36*(3), 179-192. doi: 10.1080/16506070701381863

Saarni, S. I., Suvisaari, J., Sintonen, H., Pirkola, S., Koskinen, S., Aromaa, A., & Lonnqvist, J. (2007). Impact of psychiatric disorders on health-related quality of life: general population survey. *Br J Psychiatry, 190*, 326-332. doi: 10.1192/bjp.bp.106.025106

Sakurai, K., Nishi, A., Kondo, K., Yanagida, K., & Kawakami, N. (2011). Screening performance of K6/K10 and other screening instruments for mood and anxiety disorders in Japan. *Psychiatry Clin Neurosci, 65*(5), 434-441. doi: 10.1111/j.1440-1819.2011.02236.x

Shimazu, A., Schaufeli, W. B., Kosugi, S., Suzuki, A., Nashiwa, H., Kato, A., . . . Goto, R. (2008). Work engagement in Japan: Validation of the Japanese version of the Utrecht Work Engagement Scale. *Applied Psychology-an International Review-Psychologie Appliquee-Revue Internationale, 57*(3), 510-523. doi: DOI 10.1111/j.1464-0597.2008.00333.x

Sones, W. W. D. (1944). The Comics and Instructional Method. *Journal of Educational Sociology, 18*(4), 232-240. doi: Doi 10.2307/2262696

Spek, V., Cuijpers, P., Nyklicek, I., Riper, H., Keyzer, J., & Pop, V. (2007). Internet-based cognitive behaviour therapy for symptoms of depression and anxiety: a meta-analysis. *Psychol Med, 37*(3), 319-328. doi: 10.1017/S0033291706008944

Tajima, M., Akiyama, T., Numa, H., Kawamura, Y., Okada, Y., Sakai, Y., . . . Power, M. J. (2007). Reliability and validity of the Japanese version of the 24-item Dysfunctional Attitude Scale. *Acta Neuropsychiatrica, 19*(6), 362-367. doi: DOI 10.1111/j.1601-5215.2007.00203.x

Titov, N., Andrews, G., & Sachdev, P. (2010). Computer-delivered cognitive behavioural therapy: effective and getting ready for dissemination. *F1000 Med Rep, 2*. doi: 10.3410/M2-49

van der Klink, J. J., Blonk, R. W., Schene, A. H., & van Dijk, F. J. (2001). The benefits of interventions for work-related stress. *Am J Public Health, 91*(2), 270-276.

World Health Organization. Department of Mental Health and Substance Abuse. (2001). *Atlas: Mental Health Resources in the World 2001*. Geneva: World Health Organization.

World Health Organization. Department of Mental Health and Substance Abuse. (2005). *Mental health atlas 2005* (Rev ed.). Geneva: World Health Organization.
